# Supplementary material for: An activity theory-based exploration of “Eyeland”, a task-based serious game for EFL visually impaired students
Source: PeerJ Comput Sci. 2025 Apr 23;11:e2631. doi: 10.7717/peerj-cs.2631 (PMC12190295; doi:10.7717/peerj-cs.2631)
Supplement: Supplemental Information 1 — These were organized to ensure that the questions were informed by activity theory, include the accessibility aspects of the context and explore the level of awareness regarding inclusion before and after implementation. The questionnaire was validated by Bligh & Coyle (2013), who evaluated a presentation tool via activity theory. [file peerj-cs-11-2631-s001.docx]

Activity theory-based interviews with teachers

These were organized to ensure that the questions were informed by activity theory, include the accessibility aspects of the context and explore the level of awareness regarding inclusion before and after implementation.

*Activity theory-based questions*

| Describe a regular English class. Phases/stages | Mediation (Tools) |
| --- | --- |
| What resources are available to teach your traditional lessons? | Mediation (Tools) |
| What are the characteristics of the classroom where you teach English? | Mediation (Tools) |
| What tools and materials are accessible? | Mediation (Tools) |
| What procedures do you follow by supporting the visually impaired students? | Object |
| Have you used mobile learning in your classroom? | Mediation (Tools) |
| Have you used task-based learning in class? How frequently do you use it? | Outcomes |
| What are the common problems and limitations that you encounter in your traditional lessons? | Object |
| What are the roles of the other students while interacting with the visually impaired students? | Community of practice |
| How does the school support the process with visually impaired students? | Division of labor |
| What skills are commonly reinforced in a regular class? | Object |
| What are your expectations of the use of an app in your class? | Outcomes |
| Do you believe that a serious game might work in terms of support for learning? Why or why not | Object |

*Note*. The questionnaire was validated by Bligh & Coyle (2013), who evaluated a presentation tool via activity theory.

*Post-implementation interviews with teachers*

| What were your impressions of Eyeland’s use within the classes? | Mediation (Tools) |
| --- | --- |
| What worked well with how students used Eyeland? | Subject |
| What did not work well about how students used Eyeland? | Subject |
| What do you like about the Eyeland technology? | Mediation (Tools) |
| What do you think are the current technical constraints of Eyeland? | Mediation (Tools) |
| What would you like Eyeland to be able to do that does not currently seem possible? | Mediation (Tools) |
| Would you like to use Eyeland in the future? If so, what for? | Outcomes |
| What impact does Eyeland have on lessons? | Object |
| What aspects are necessary to re mediate the lessons through the app? | Rules |

*Note*. The questionnaire was validated by Bligh & Coyle (2013), who evaluated a presentation tool via activity theory.
