# Supplementary material for: An activity theory-based exploration of “Eyeland”, a task-based serious game for EFL visually impaired students
Source: PeerJ Comput Sci. 2025 Apr 23;11:e2631. doi: 10.7717/peerj-cs.2631 (PMC12190295; doi:10.7717/peerj-cs.2631)
Supplement: Supplemental Information 2 — This table was designed based on the proposal of Bybee et al. (2006).It illustrates the tools and the task-based learning elements and the way it was used in class. [file peerj-cs-11-2631-s002.docx]

*5E instructional model. Lesson plan around Eyeland app*

| *Lesson Elements* | *What does it look like?* | *What tools can I use?* |
| --- | --- | --- |
| ***Engage*** | - Brainstorm - Ask questions. - Access prior knowledge - Pique their interest | Magnified materials, Braille, Audio Books  Recorders, Special needs assistant, Realia |
| ***Explore*** | - Conduct research - Explore teacher-curated resources - Offline task - Crowdsource | Pre- task Eyeland |
| ***Explain*** | - Live presentation in class or in video conferencing session - Recorded video explanations | Pre- task Eyeland  Magnified materials, Braille, Audio Books  Recorders, Special needs assistant, Realia, AI powered voice |
| ***Elaborate*** | - Make connections. - Apply the learning to new or novel situations - Explain how - Student –centered study materials and resources | During task-Eyeland |
| ***Evaluate*** | - Assessments - Video reflections - Digital exit tickets | Post -task Eyeland |

*Note*. This table was designed based on the proposal of Bybee et al. (2006).
