# Supplementary material for: An activity theory-based exploration of “Eyeland”, a task-based serious game for EFL visually impaired students
Source: PeerJ Comput Sci. 2025 Apr 23;11:e2631. doi: 10.7717/peerj-cs.2631 (PMC12190295; doi:10.7717/peerj-cs.2631)
Supplement: Supplemental Information 4 — The thematic analysis coding provides a structured guide to understanding how data were categorized into themes and subcategories, ensuring clarity and consistency in the analysis. Each category and subcategory represents a specific aspect of the data, derived from observations, interviews, and focus groups. [file peerj-cs-11-2631-s004.pdf]

Thematic analysis phases draft

Phase	Action	Example Outcome
**Initial Coding**	Assign descriptive labels to data extracts to summarize their content.	- "Intrinsic Motivation" coded as *desire to engage with Eyeland*.
		- "Technical Difficulties" coded as *issues with software or hardware usability*.
**Searching for Themes**	Group related codes into candidate themes, organizing them hierarchically.	- *Engagement*: Includes codes such as *desire to engage, participation,* and *enjoyment*.
		- *Accessibility Issues*: Includes *technical issues* and *interface usability*.
**Reviewing Themes**	Verify themes against the dataset to ensure relevance and coherence.	- Refining *Engagement* to emphasize active participation and its link to intrinsic motivation.
		- Merging *Technical Difficulties* and *Support Limitations* into a broader theme: *Accessibility Barriers*.
**Refining Themes**	Clearly define each theme, ensuring distinctiveness and alignment with research questions.	- *Engagement*: Captures motivational, emotional, and interactive aspects.
		- *Accessibility Barriers*: Focuses on usability challenges and solutions.
		- *Impact on Teaching*: Highlights EFL improvements and integration challenges.

Phase	Action	Example Outcome
**Producing the Report**	Create a detailed narrative explaining each theme, supported by data extracts and subcategories.	- *Engagement*: Students reported high levels of excitement (*“A game? That sounds fun!”*).
		- *Accessibility Barriers*: Students highlighted issues with screen readers and insufficient training.
		- *Impact on Teaching*: Teachers discussed the need for better curriculum alignment with Eyeland's design.

Example Detailed Subsections from the Report

1. **Engagement**
   - **Intrinsic Motivation**: "Students expressed a strong desire to engage with Eyeland due to its gamified structure."
   - **Active Participation**: "Participants reported active roles in team-based challenges, fostering collaboration and focus."
2. **Accessibility Barriers**
   - **Technical Difficulties**: "Poor internet connectivity hindered seamless gameplay for several students."
   - **User-Friendly Interface Design**: "While intuitive for many, visually impaired students faced challenges without appropriate adjustments."
3. **Impact on Teaching**
   - **Curriculum Adaptability**: "Teachers noted difficulty aligning Eyeland activities with standardized lesson plans, citing increased preparation time."

Thematic analysis final coding

Category	Subcategory	Description
**Engagement**	**Intrinsic Motivation**	Students' internal desire to participate and learn through the 'Eyeland' game.
	**Active Participation**	Level of students' involvement and activity during the game's tasks.
	**Enjoyment and Fun**	Feelings of pleasure and entertainment experienced while interacting with the game.
	**Social Interaction**	Collaboration and communication with other students while using the application.
	**Attention and Focus**	Students' ability to stay concentrated on the game's tasks.
	**Repeat Usage**	Frequency with which students choose to play 'Eyeland' again.
	**Positive Feedback**	Favorable comments from students about their experience with the game.
**Accessibility Issues**	**Technical Difficulties**	Hardware or software issues affecting students' use of the game.
	**Compatibility with Assistive Technologies**	Integration of the game with support devices and software for visually impaired students (e.g., screen readers, Braille displays).
	**Clarity of Auditory Instructions**	Ease with which students understand the verbal instructions provided by the game.
	**Cognitive Load**	Mental effort required to navigate and complete tasks in the game.
	**User-Friendly Interface Design**	Accessibility and usability of the game's navigation and control elements for visually impaired users.
	**Physical Limitations**	Challenges related to visual impairment that affect interaction with the game (e.g., tactile sensitivity).
	**Insufficient Technical Support**	Lack of assistance or resources to resolve technical issues encountered by users.
**Impact on Teaching**	**Improvement in EFL Skills**	Progress in listening, speaking, reading, and writing skills in English as a Foreign Language (EFL).

Category	Subcategory	Description
	**Application of Theory to Practice**	How the game facilitates understanding and application of theoretical concepts in practical learning situations.
	**Increased Autonomy**	Students' ability to learn and solve problems independently through the game.
	**Curriculum Adaptability**	Ease of integrating 'Eyeland' into the curriculum and aligning it with existing educational goals.
	**Changes in Teaching Methodology**	Modifications in teaching strategies by educators when incorporating the game into their classes.
	**Formative Assessment**	Use of the game as a tool to monitor and evaluate students' progress in real-time.
	**Teacher Feedback**	Teachers' opinions and perceptions about the game's effectiveness and usefulness in the teaching-learning process.
**Emerging Data**	**Overall Satisfaction**	General level of satisfaction of students with the experience of using 'Eyeland'.
	**Cultural or Linguistic Barriers**	Obstacles related to the game's cultural or linguistic content that may affect students' understanding or interest.
	**Personalized Learning**	Ability to adapt the game to students' individual needs and preferences.
	**Confidence in Technological Skills**	Students' confidence and competence in using digital tools for learning.
	**Improvement Suggestions**	Recommendations from users for future updates or improvements to the game.

Category	Subcategory	Quotes from Interviews
**Engagement**	**Active Participation**	"The interactive nature of the app kept them engaged, especially the visually impaired students who often feel left out during traditional lessons."
	**Enjoyment and Fun**	"I think Eyeland helped make lessons more interactive and fun. The visually impaired students enjoyed it because they could follow along more easily with the screen reader feature."
	**Positive Feedback**	"The feedback system was great. Students could immediately see where they made mistakes and fix them."
**Accessibility Issues**	**Technical Difficulties**	"The technical constraints were a big issue. The app sometimes froze due to poor internet, and not all students were comfortable using it."
		"The internet is the biggest problem. Without a stable connection, the app doesn't work properly, and it disrupts the lesson."
	**Compatibility with Assistive Technologies**	"The app has features like the screen reader that make it easy for visually impaired students to participate."
	**User-Friendly Interface Design**	"It could work if designed properly. Games would definitely engage students more, but only if they're accessible and easy to use."
	**Insufficient Technical Support**	"If we get more training, I believe the app could become an important tool in our teaching. But right now, it's hard to fully implement it without more support from the school."
**Impact on Teaching**	**Curriculum Adaptability**	"It doesn't match our syllabus perfectly, so I feel like I'm spending too much time adapting lessons."

	**Changes in Teaching Methodology**	"Gamification would help here, but the tools we use aren't sufficient for that. I've heard about apps that make learning more interactive, but I've never used them because I don't know how."
	**Teacher Feedback**	"The first thing is training. Teachers need to feel confident using the app, and we need lesson plans that match our syllabus."
**Emerging Data**	**Overall Satisfaction**	"Students seem to enjoy learning more when they're using the app."
	**Confidence in Technological Skills**	"It's my first time using apps."
		"I don't know how to fully support them using technology, especially since I'm not familiar with apps or digital tools myself."
	**Improvement Suggestions**	"If it could work offline, that would solve so many problems. Also, more pre-made lesson plans would be great—especially ones that align with our syllabus."

Engagement

Subcategory	Quote from observation
Intrinsic Motivation	"Super Hearing! That sounds awesome. I can finally use the screen reader to help my group. I have a tablet at home."
Active Participation	"The app asks: What does Otto eat?"
Enjoyment and Fun	"A game? That sounds fun!"
Social Interaction	"Great teamwork! Keep following the app's prompts and remember to support each other."
Attention and Focus	"I'm listening! The next word is 'river.' It means using scientific knowledge for practical purposes."
Repeat Usage	"Yes, we're right!"
Positive Feedback	"Fantastic! Now, I want everyone to open the Eyeland app. You'll find the first scenario waiting for you. It's a vocabulary challenge."

Accessibility Issues

Subcategory	Quote from Observations
**Technical Difficulties**	"The technical constraints were a big issue. The app sometimes froze due to poor internet, and not all students were comfortable using it."
**Compatibility with Assistive Technologies**	"The app has features like the screen reader that make it easy for visually impaired students to participate."
**User-Friendly Interface Design**	"It could work if designed properly. Games would definitely engage students more, but only if they're accessible and easy to use."
**Insufficient Technical Support**	"If we get more training, I believe the app could become an important tool in our teaching. But right now, it's hard to fully implement it without more support from the school."

Impact on Teaching

Subcategory	Quote from observations
**Curriculum Adaptability**	"It doesn't match our syllabus perfectly, so I feel like I'm spending too much time adapting lessons."
**Changes in Teaching Methodology**	"Gamification would help here, but the tools we use aren't sufficient for that. I've heard about apps that make learning more interactive, but I've never used them because I don't know how."
**Teacher Feedback**	"The first thing is training. Teachers need to feel confident using the app, and we need lesson plans that match our syllabus."

Emerging Data

Subcategory	Quote from observations
**Overall Satisfaction**	"Students seem to enjoy learning more when they're using the app."
**Confidence in Technological Skills**	"I don't know how to fully support them using technology, especially since I'm not familiar with apps or digital tools myself."
**Improvement Suggestions**	"If it could work offline, that would solve so many problems. Also, more pre-made lesson plans would be great—especially ones that align with our syllabus."

Emerging Data from focus groups

Subcategory	Comment
**Overall Satisfaction**	"It is complete to me."
	"In general it is quite good. It helps a lot."
**Improvement Suggestions**	"Letters can be dragged in the word-forming activity."
**Overall Satisfaction**	"Nothing. Everything is ok."
**No Feedback Provided**	"No comments."
