# Supplementary material for: An activity theory-based exploration of “Eyeland”, a task-based serious game for EFL visually impaired students
Source: PeerJ Comput Sci. 2025 Apr 23;11:e2631. doi: 10.7717/peerj-cs.2631 (PMC12190295; doi:10.7717/peerj-cs.2631)
Supplement: Supplemental Information 5 — Original file of students responses regarding the usability of the app. [file peerj-cs-11-2631-s005.pdf]

Escala de Preferencia de Videojuegos Educativos

A continuación, aparecen una serie de comentarios que describen sentimientos y opiniones con respecto a su satisfacción con algunos videojuegos. Lea cada uno de los comentarios y marca aquel con el cual te sientas más de acuerdo.

		Muy en desacuerdo	En desacuerdo	Ni en desacuerdo, ni de acuerdo	De acuerdo	Muy de acuerdo
P1	Me divierte resolver acertijos o juegos				X	
P2	Disfruto descifrar crucigramas o sopas de letras		X			
P3	Me aburre adivinar la ubicación de objetos en una imagen o mapa					X
P4	Me aburre el completar las palabras que faltan en un párrafo o frase			X		
P5	Disfruto ordenando cosas en un juego			X		
P6	Me fastidia rellenar palabras incompletas	X		X		
P7	Me angustia perder puntos en un juego					
P8	Me frustra tener que pedir ayuda cuando me atasco en una actividad					
p9	No me gusta tener un límite de intentos en una actividad					
p10	Me frustra perder puestos en una competencia					
p11	Un juego sin puntos no es interesante					
p12	Competir es más divertido que colaborar					
p13	Disfruto el tener que clasificar palabras u objetos					
p14	Me gustan las actividades donde debes relacionar objetos que pertenecen a una categoría.					

Escala de Preferencia de Videojuegos Educativos

A continuación, aparecen una serie de comentarios que describen sentimientos y opiniones con respecto a su satisfacción con algunos videojuegos. Lea cada uno de los comentarios y marca aquel con el cual te sientas más de acuerdo.

		Muy en desacuerdo	En desacuerdo	Ni en desacuerdo, ni de acuerdo	De acuerdo	Muy de acuerdo
P1	Me divierte resolver acertijos o juegos					✓
P2	Disfruto descifrar crucigramas o sopas de letras					✓
P3	Me aburre adivinar la ubicación de objetos en una imagen o mapa			✓		
P4	Me aburre el completar las palabras que faltan en un párrafo o frase		✓			
P5	Disfruto ordenando cosas en un juego				✓	
P6	Me fastidia rellenar palabras incompletas	✓				
P7	Me angustia perder puntos en un juego			✓		
P8	Me frustra tener que pedir ayuda cuando me atasco en una actividad					✓
p9	No me gusta tener un límite de intentos en una actividad	✓				
p10	Me frustra perder puestos en una competencia					✓
p11	Un juego sin puntos no es interesante					✓
p12	Competir es más divertido que colaborar			✓		
p13	Disfruto el tener que clasificar palabras u objetos					✓
p14	Me gusta las actividades donde debes relacionar objetos que pertenecen a una categoría.					✓

Escala de Preferencia de Videojuegos Educativos

A continuación, aparecen una serie de comentarios que describen sentimientos y opiniones con respecto a su satisfacción con algunos videojuegos. Lea cada uno de los comentarios y marca aquel con el cual te sientas más de acuerdo.

		Muy en desacuerdo	En desacuerdo	Ni en desacuerdo, ni de acuerdo	De acuerdo	Muy de acuerdo
P1	Me divierte resolver acertijos o juegos				X	
P2	Disfruto descifrar crucigramas o sopas de letras				X	
P3	Me aburre adivinar la ubicación de objetos en una imagen o mapa		X			
P4	Me aburre el completar las palabras que faltan en un párrafo o frase		X			
P5	Disfruto ordenando cosas en un juego				X	
P6	Me fastidia rellenar palabras incompletas				X	
P7	Me angustia perder puntos en un juego				X	
P8	Me frustra tener que pedir ayuda cuando me atasco en una actividad				X	
p9	No me gusta tener un límite de intentos en una actividad		X			
p10	Me frustra perder puestos en una competencia				X	
p11	Un juego sin puntos no es interesante					
p12	Competir es más divertido que colaborar		X			
p13	Disfruto el tener que clasificar palabras u objetos		X			
p14	Me gusta las actividades donde debes relacionar objetos que pertenecen a una categoría.		X			

Escala de Preferencia de Videojuegos Educativos

A continuación, aparecen una serie de comentarios que describen sentimientos y opiniones con respecto a su satisfacción con algunos videojuegos. Lea cada uno de los comentarios y marca aquel con el cual te sientas más de acuerdo.

		Muy en desacuerdo	En desacuerdo	Ni en desacuerdo, ni de acuerdo	De acuerdo	Muy de acuerdo
P1	Me divierte resolver acertijos o juegos				X	
P2	Disfruto descifrar crucigramas o sopas de letras					X
P3	Me aburre adivinar la ubicación de objetos en una imagen o mapa			X		
P4	Me aburre el completar las palabras que faltan en un párrafo o frase		X			
P5	Disfruto ordenando cosas en un juego					X
P6	Me fastidia rellenar palabras incompletas			X		
P7	Me angustia perder puntos en un juego				X	
P8	Me frustra tener que pedir ayuda cuando me atasco en una actividad		X			
p9	No me gusta tener un límite de intentos en una actividad				X	
p10	Me frustra perder puestos en una competencia				X	
p11	Un juego sin puntos no es interesante			X		
p12	Competir es más divertido que colaborar		X			
p13	Disfruto el tener que clasificar palabras u objetos				X	
p14	Me gusta las actividades donde debes relacionar objetos que pertenecen a una categoría.				X	

Escala de Preferencia de Videojuegos Educativos

A continuación, aparecen una serie de comentarios que describen sentimientos y opiniones con respecto a su satisfacción con algunos videojuegos. Lea cada uno de los comentarios y marca aquel con el cual te sientas más de acuerdo.

		Muy en desacuerdo	En desacuerdo	Ni en desacuerdo, ni de acuerdo	De acuerdo	Muy de acuerdo
P1	Me divierte resolver acertijos o juegos				X	
P2	Disfruto descifrar crucigramas o sopas de letras					X
P3	Me aburre adivinar la ubicación de objetos en una imagen o mapa				X	
P4	Me aburre el completar las palabras que faltan en un párrafo o frase				X	
P5	Disfruto ordenando cosas en un juego					X
P6	Me fastidia rellenar palabras incompletas		X			
P7	Me angustia perder puntos en un juego					X
P8	Me frustra tener que pedir ayuda cuando me atasco en una actividad			X		
p9	No me gusta tener un límite de intentos en una actividad			X		
p10	Me frustra perder puestos en una competencia		X			
p11	Un juego sin puntos no es interesante			X		
p12	Competir es más divertido que colaborar				X	
p13	Disfruto el tener que clasificar palabras u objetos			X		
p14	Me gusta las actividades donde debes relacionar objetos que pertenecen a una categoría.			X		

Escala de Preferencia de Videojuegos Educativos

A continuación, aparecen una serie de comentarios que describen sentimientos y opiniones con respecto a su satisfacción con algunos videojuegos. Lea cada uno de los comentarios y marca aquel con el cual te sientas más de acuerdo.

		Muy en desacuerdo	En desacuerdo	Ni en desacuerdo, ni de acuerdo	De acuerdo	Muy de acuerdo
P1	Me divierte resolver acertijos o juegos				✓	
P2	Disfruto descifrar crucigramas o sopas de letras				✓	
P3	Me aburre adivinar la ubicación de objetos en una imagen o mapa			✓		
P4	Me aburre el completar las palabras que faltan en un párrafo o frase			✓		
P5	Disfruto ordenando cosas en un juego			✓		
P6	Me fastidia rellenar palabras incompletas			✓		
P7	Me angustia perder puntos en un juego				✓	
P8	Me frustra tener que pedir ayuda cuando me atasco en una actividad				✓	
p9	No me gusta tener un límite de intentos en una actividad				✓	
p10	Me frustra perder puestos en una competencia				✓	
p11	Un juego sin puntos no es interesante			✓		
p12	Competir es más divertido que colaborar			✓		
p13	Disfruto el tener que clasificar palabras u objetos			✓		
p14	Me gusta las actividades donde debes relacionar objetos que pertenecen a una categoría.			✓		

Escala de Preferencia de Videojuegos Educativos

A continuación, aparecen una serie de comentarios que describen sentimientos y opiniones con respecto a su satisfacción con algunos videojuegos. Lea cada uno de los comentarios y marca aquel con el cual te sientas más de acuerdo.

		Muy en desacuerdo	En desacuerdo	Ni en desacuerdo, ni de acuerdo	De acuerdo	Muy de acuerdo
P1	Me divierte resolver acertijos o juegos				X	
P2	Disfruto descifrar crucigramas o sopas de letras				X	
P3	Me aburre adivinar la ubicación de objetos en una imagen o mapa		X			
P4	Me aburre el completar las palabras que faltan en un párrafo o frase		X			
P5	Disfruto ordenando cosas en un juego				X	
P6	Me fastidia rellenar palabras incompletas		X			
P7	Me angustia perder puntos en un juego		X			
P8	Me frustra tener que pedir ayuda cuando me atasco en una actividad				X	
p9	No me gusta tener un límite de intentos en una actividad		X			
p10	Me frustra perder puestos en una competencia		X			
p11	Un juego sin puntos no es interesante				X	
p12	Competir es más divertido que colaborar		X			
p13	Disfruto el tener que clasificar palabras u objetos				X	
p14	Me gustan las actividades donde debes relacionar objetos que pertenecen a una categoría.				X	

Escala de Preferencia de Videojuegos Educativos

A continuación, aparecen una serie de comentarios que describen sentimientos y opiniones con respecto a su satisfacción con algunos videojuegos. Lea cada uno de los comentarios y marca aquel con el cual te sientas más de acuerdo.

		Muy en desacuerdo	En desacuerdo	Ni en desacuerdo, ni de acuerdo	De acuerdo	Muy de acuerdo
P1	Me divierte resolver acertijos o juegos					
P2	Disfruto descifrar crucigramas o sopas de letras					
P3	Me aburre adivinar la ubicación de objetos en una imagen o mapa					
P4	Me aburre el completar las palabras que faltan en un párrafo o frase					
P5	Disfruto ordenando cosas en un juego					
P6	Me fastidia rellenar palabras incompletas					
P7	Me angustia perder puntos en un juego					
P8	Me frustra tener que pedir ayuda cuando me atasco en una actividad					
p9	No me gusta tener un límite de intentos en una actividad					
p10	Me frustra perder puestos en una competencia					
p11	Un juego sin puntos no es interesante					
p12	Competir es más divertido que colaborar					
p13	Disfruto el tener que clasificar palabras u objetos					
p14	Me gustan las actividades donde debes relacionar objetos que pertenecen a una categoría.					

Escala de Preferencia de Videojuegos Educativos

A continuación, aparecen una serie de comentarios que describen sentimientos y opiniones con respecto a su satisfacción con algunos videojuegos. Lea cada uno de los comentarios y marca aquel con el cual te sientas más de acuerdo.

		Muy en desacuerdo	En desacuerdo	Ni en desacuerdo, ni de acuerdo	De acuerdo	Muy de acuerdo
P1	Me divierte resolver acertijos o juegos				✓	
P2	Disfruto descifrar crucigramas o sopas de letras			✓		
P3	Me aburre adivinar la ubicación de objetos en una imagen o mapa				✓	
P4	Me aburre el completar las palabras que faltan en un párrafo o frase			✓		
P5	Disfruto ordenando cosas en un juego				✓	
P6	Me fastidia rellenar palabras incompletas				✓	
P7	Me angustia perder puntos en un juego			✓		
P8	Me frustra tener que pedir ayuda cuando me atasco en una actividad				✓	
p9	No me gusta tener un límite de intentos en una actividad				✓	
p10	Me frustra perder puestos en una competencia				✓	
p11	Un juego sin puntos no es interesante			✓		
p12	Competir es más divertido que colaborar			✓		
p13	Disfruto el tener que clasificar palabras u objetos		✓			
p14	Me gusta las actividades donde debes relacionar objetos que pertenecen a una categoría.				✓	

Escala de Preferencia de Videojuegos Educativos

A continuación, aparecen una serie de comentarios que describen sentimientos y opiniones con respecto a su satisfacción con algunos videojuegos. Lea cada uno de los comentarios y marca aquel con el cual te sientas más de acuerdo.

		Muy en desacuerdo	En desacuerdo	Ni en desacuerdo, ni de acuerdo	De acuerdo	Muy de acuerdo
P1	Me divierte resolver acertijos o juegos					✓
P2	Disfruto descifrar crucigramas o sopas de letras					✓
P3	Me aburre adivinar la ubicación de objetos en una imagen o mapa	✓				
P4	Me aburre el completar las palabras que faltan en un párrafo o frase	✓				
P5	Disfruto ordenando cosas en un juego				✓	
P6	Me fastidia rellenar palabras incompletas	✓				
P7	Me angustia perder puntos en un juego			✓		
P8	Me frustra tener que pedir ayuda cuando me atasco en una actividad		✓			
p9	No me gusta tener un límite de intentos en una actividad		✓			
p10	Me frustra perder puestos en una competencia			✓		
p11	Un juego sin puntos no es interesante		✓			
p12	Competir es más divertido que colaborar	✓				
p13	Disfruto el tener que clasificar palabras u objetos				✓	
p14	Me gusta las actividades donde debes relacionar objetos que pertenecen a una categoría.				✓	

Escala de Preferencia de Videojuegos Educativos

A continuación, aparecen una serie de comentarios que describen sentimientos y opiniones con respecto a su satisfacción con algunos videojuegos. Lea cada uno de los comentarios y marca aquel con el cual te sientas más de acuerdo.

		Muy en desacuerdo	En desacuerdo	Ni en desacuerdo, ni de acuerdo	De acuerdo	Muy de acuerdo
P1	Me divierte resolver acertijos o juegos					✓
P2	Disfruto descifrar crucigramas o sopas de letras					✓
P3	Me aburre adivinar la ubicación de objetos en una imagen o mapa		✓			
P4	Me aburre el completar las palabras que faltan en un párrafo o frase			✓		
P5	Disfruto ordenando cosas en un juego					✓
P6	Me fastidia rellenar palabras incompletas		✓			
P7	Me angustia perder puntos en un juego				✓	
P8	Me frustra tener que pedir ayuda cuando me atasco en una actividad					✓
p9	No me gusta tener un límite de intentos en una actividad		✓			
p10	Me frustra perder puestos en una competencia				✓	
p11	Un juego sin puntos no es interesante			✓		
p12	Competir es más divertido que colaborar	✓				
p13	Disfruto el tener que clasificar palabras u objetos				✓	
p14	Me gustan las actividades donde debes relacionar objetos que pertenecen a una categoría.				✓	

Escala de Preferencia de Videojuegos Educativos

A continuación, aparecen una serie de comentarios que describen sentimientos y opiniones con respecto a su satisfacción con algunos videojuegos. Lea cada uno de los comentarios y marca aquel con el cual te sientas más de acuerdo.

		Muy en desacuerdo	En desacuerdo	Ni en desacuerdo, ni de acuerdo	De acuerdo	Muy de acuerdo
P1	Me divierte resolver acertijos o juegos				✓	
P2	Disfruto descifrar crucigramas o sopas de letras			✓		
P3	Me aburre adivinar la ubicación de objetos en una imagen o mapa		✓			
P4	Me aburre el completar las palabras que faltan en un párrafo o frase			✓		
P5	Disfruto ordenando cosas en un juego				✓	
P6	Me fastidia rellenar palabras incompletas					✓
P7	Me angustia perder puntos en un juego					✓
P8	Me frustra tener que pedir ayuda cuando me atasco en una actividad					✓
p9	No me gusta tener un límite de intentos en una actividad					✓
p10	Me frustra perder puestos en una competencia					✓
p11	Un juego sin puntos no es interesante					✓
p12	Competir es más divertido que colaborar					✓
p13	Disfruto el tener que clasificar palabras u objetos				✓	
p14	Me gusta las actividades donde debes relacionar objetos que pertenecen a una categoría.			✓		
