# Supplementary material for: An activity theory-based exploration of “Eyeland”, a task-based serious game for EFL visually impaired students
Source: PeerJ Comput Sci. 2025 Apr 23;11:e2631. doi: 10.7717/peerj-cs.2631 (PMC12190295; doi:10.7717/peerj-cs.2631)
Supplement: Supplemental Information 6 — Answers shared by students while taking the pre-test. [file peerj-cs-11-2631-s006.pdf]

Test 1 Speaking

Name: ____________________

Date: ____________________

Part 1 (3-4 minutes)

Phase 1
Interlocutor

To both candidates	Good morning / afternoon / evening.<br>Can I have your mark sheets, please?	
	Hand over the mark sheets to the Assessor.	
	I'm …………, and this is ………. .	
To Candidate A	What's your name?	
To Candidate B	And what's your name?	

		**Back-up prompts**
	**B**, do you work or are you a student?	Do you work? Do you study? Are you a student?
For UK, ask	Where do you come from?	Are you from (Spain, etc.)?
For Non-UK, ask	Where do you live?	Do you live in … (name of district / town etc.)?
	Thank you.	
	**A,** do you work or are you a student?	Do you work? Do you study? Are you a student?
For UK, ask	Where do you come from?	Are you from (Spain, etc.)?
For Non-UK, ask	Where do you live?	Do you live in … (name of district / town etc.)?
	Thank you.	

Phase 2
Interlocutor

Now, let's talk about **friends**.

	Back-up prompts
**A**, how often do you see your friends?	Do you see your friends every day?
What do you like doing with your friends?	Do you like going to the cinema?
**B**, where do your friends live?	Do your friends live near you?
When do you see your friends?	Do you see your friends at weekends?

Extended Response
Now **A**, please tell me something about one of your friends.

Back-up questions
Do you like your friend?
Where did you meet your friend?
Did you see your friends last weekend?

Interlocutor

Now, let's talk about **home**.

	Back-up prompts
**B**, who do you live with?	Do you live with your family?
How many bedrooms are there in your house / flat?	Are there three bedrooms in your house / flat?
**A**, where do you watch TV at home?	Do you watch TV in the kitchen?
What's your favourite room in the house?	Is your bedroom your favourite room?

Extended Response
Now, **B**, please tell me something about the things you like doing at home, at the weekends.

Back-up questions
Do you like cooking at the weekends?
Do you play computer games at the weekends?
What did you do at home, last weekend?

Part 2 (5-6 minutes)

Phase 1
Interlocutor
3-4 minutes

Now, in this part of the test you are going to talk together.

*Place **Part 2** booklet, open at **Task 2a**, in front of candidates.*

Here are some pictures that show **different places to eat**.

Do you like these different places to eat? Say why or why not. I'll say that again.

Do you like these different places to eat? Say why or why not.

All right? Now, talk together.

Candidates

....

Allow a minimum of 1 minute (maximum of 2 minutes) before moving on to the following questions.

Interlocutor / Candidates
Use as appropriate. Ask each candidate at least one question.

Do you think…
….eating on the beach is fun?
… eating in restaurants is expensive?
….eating at home is boring?
… eating at college/work is cheap?
… eating in the park is nice?

Optional prompt
Why?/Why not?

What do **you** think?

Interlocutor

So, **A**, which of these places to eat do you like best?
And you, **B**, which of these places do you like best?

Thank you. (Can I have the booklet, please?) *Retrieve **Part 2** booklet.*

Phase 2

Interlocutor
Allow up to 2 minutes

Now, do you prefer eating with friends or family, **B**? (Why?)

And what about you, **A**? (Do you prefer eating with friends or family?) (Why?)

Do you prefer eating at home or in a restaurant, **A**? (Why?)

And you, **B** (Do you prefer eating at home or in a restaurant?) (Why?)

Thank you. That is the end of the test.

Do you like these different places to eat?

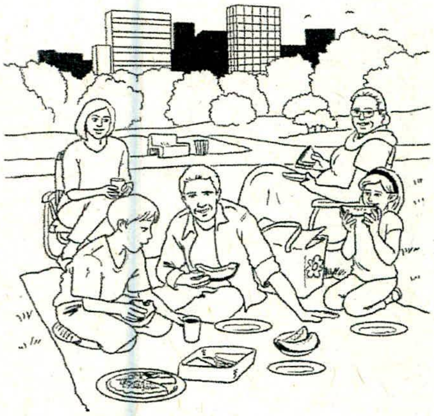

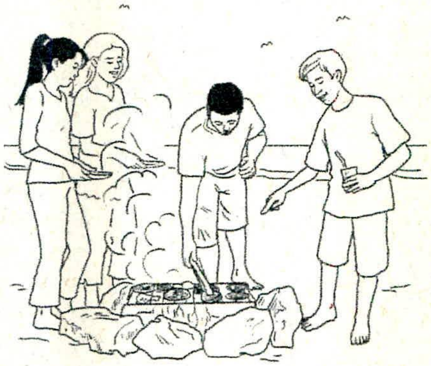

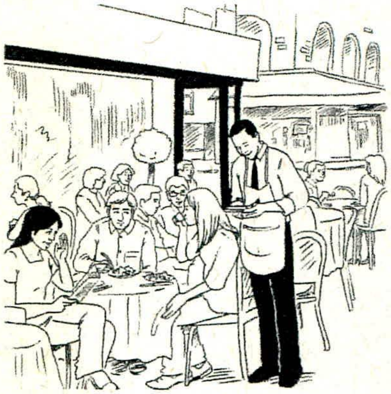

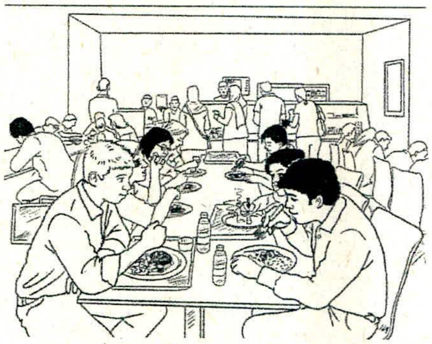

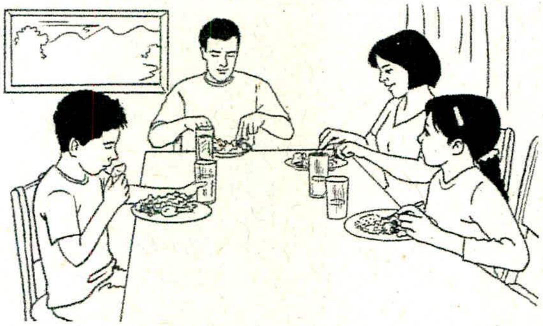

Part 1 (3-4 minutes)

Phase 1<br>**Interlocutor**	
To both candidates	Good morning / afternoon / evening.<br>Can I have your mark sheets, please?<br><br>*Hand over the mark sheets to the Assessor.*<br><br>I'm ...., and this is ....
To Candidate A	What's your name?
To Candidate B	And what's your name?

		**Back-up prompts**
	**B**, do you work or are you a student?	Do you work? Do you study? Are you a student?
For UK, ask	Where do you come from?	Are you from (Spain, etc.)?
For Non-UK, ask	Where do you live?	Do you live in ... (name of district / town etc.)?
	Thank you.	
	**A,** do you work or are you a student?	Do you work? Do you study? Are you a student?
For UK, ask	Where do you come from?	Are you from (Spain, etc.)?
For Non-UK, ask	Where do you live?	Do you live in ... (name of district / town etc.)?
	Thank you.	

Phase 2
Interlocutor

Now, let's talk about **music**.

Back-up prompts

A, how often do you listen to music? — Do you listen to music every day?

What music do you like best? — Do you like rock music?

B, what is your favourite instrument? — Do you like the piano?

Where do you like listening to music? — Do you like going to concerts?

Extended Response
Now **A**, please tell me something about your favourite singer or group?

Back-up questions
Where is your favourite singer from?
Why do you like them?
Do your friends like them too?

Interlocutor

Now, let's talk about **shopping**.

Back-up prompts

B, where do you like to go shopping? — Do you like to go to shopping centres?

What do you like to buy with your money? — Do you like to buy clothes with your money?

A, who do you like to go shopping with? — Do you like to go shopping with your friends?

What can you buy near your house? — Can you buy food near your house?

Extended Response
Now, **B**, please tell me something about presents you buy for your friends.

Back-up questions
Where do you buy presents?
Do you like giving presents?
Have you bought a present recently?

Part 2 (5-6 minutes)

Phase 1

Interlocutor
3-4 minutes

Now, in this part of the test you are going to talk together.

*Place **Part 2** booklet, open at **Task 2b**, in front of candidates.*

Here are some pictures that show **different holidays**.

Do you like these different holidays? Say why or why not. I'll say that again.

Do you like these different holidays? Say why or why not.

All right? Now, talk together.

Candidates

....

Allow a minimum of 1 minute (maximum 2 minutes) before moving on to the following questions.

Interlocutor / Candidates
Use as appropriate. Ask each candidate at least one question.

Do you think....
...beach holidays are fun?
...city holidays are interesting?
...camping holidays are exciting?
...walking holidays are expensive?
...holidays in the mountain are boring?

Optional prompt
Why?/Why not?

What do **you** think?

Interlocutor

So, **A**, which of these holidays do you like best?
And you, **B**, which of these holidays do you like best?

Thank you. (Can I have the booklet, please?) *Retrieve **Part 2** booklet.*

Phase 2

Interlocutor
Allow up to 2 minutes

Now, do you prefer to go on holidays with your friends or with your family, **B**? (Why?)

And what about you, **A**? (Do you prefer to go on holidays with your friends or with your family?) (Why?)

Which country would you like to visit in the future, **A**? (Why?)

And you, **B**? (Which country would you like to visit in the future?) (Why?)

Thank you. That is the end of the test.

Do you like these different holidays?

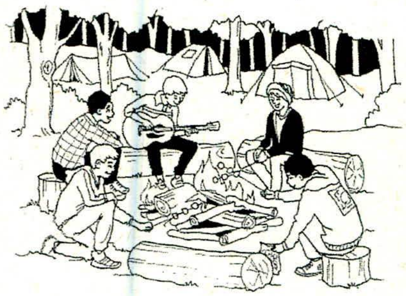

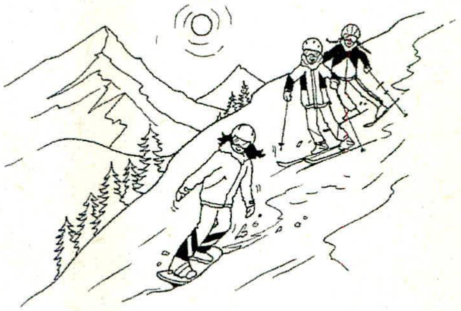

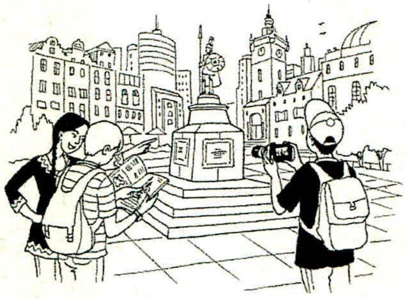

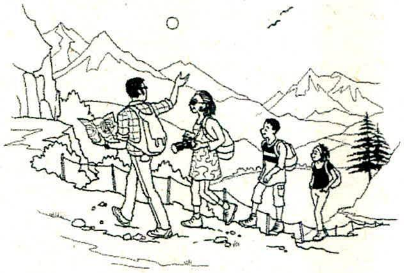

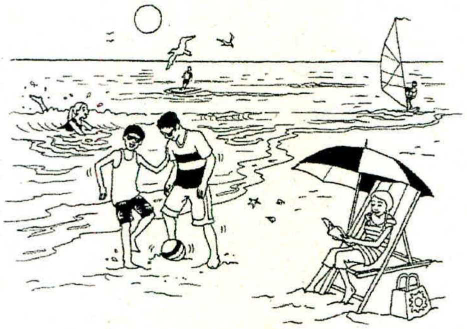

CAMBRIDGE
English

Kelvin Enazo

Key

Listening

Sample Test

TIME Approximately 35 minutes (including 6 minutes' transfer time)

INSTRUCTIONS TO CANDIDATES

Do not open this question paper until you are told to do so.

Write your name, centre number and candidate number on your answer sheet if they are not already there.

Listen to the instructions for each part of the paper carefully.

Answer all the questions.

While you are listening, write your answers on the question paper.

You will have 6 minutes at the end of the test to copy your answers onto the separate answer sheet. Use a pencil.

At the end of the test, hand in both this question paper and your answer sheet.

INFORMATION FOR CANDIDATES

There are five parts to the test.

Each question carries one mark.

You will hear each piece twice.

For each part of the test there will be time for you to look through the questions and time for you to check your answers.

Copyright © Cambridge University Press & Assessment 2022

Part 1

Questions 1 – 5

For each question, choose the correct answer.

---

1 Where will Claire meet Alex?

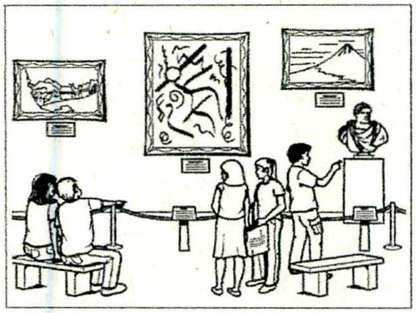

A

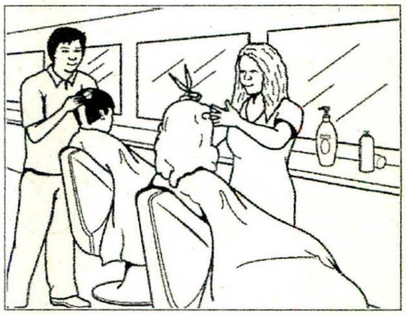

B

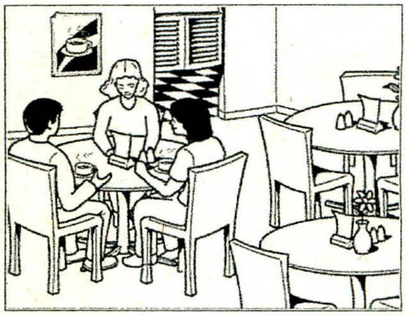

C

2 What time should the man telephone again?

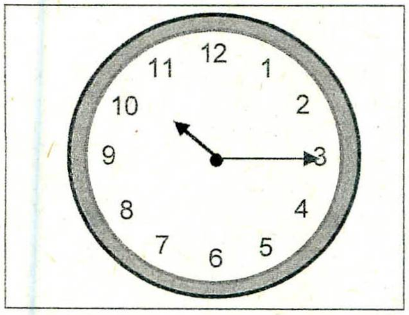

A

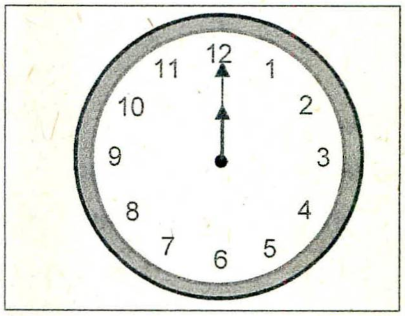

B

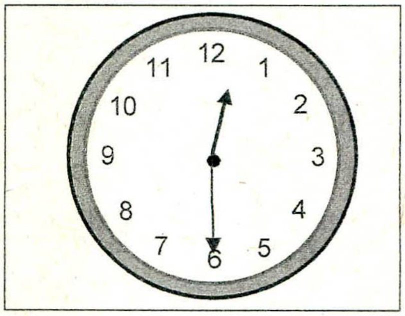

C

3 When are they going to have the party?

A	B	C
July 11	July 18	July 25

4 What was the weather like on the picnic?

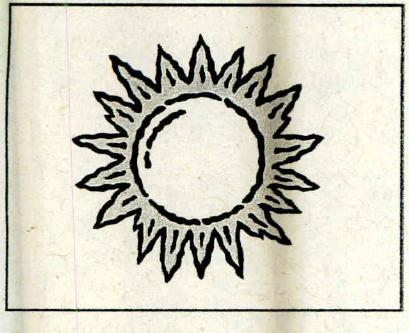

A

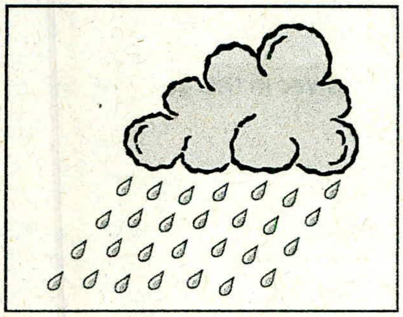

B

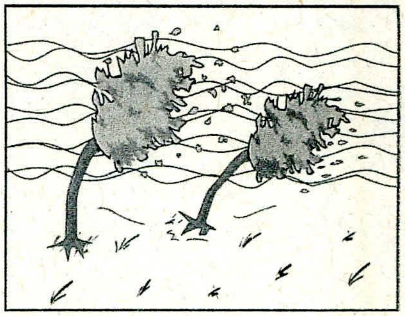

C

5 How much are the shorts?

A	B	C
£5	£15	£20

Part 2

Questions 6 – 10

For each question, write the correct answer in the gap. Write **one word** or a **number** or a **date** or a **time**.

You will hear a teacher talking to a group of students about summer jobs.

Jobs for students
with *Sunshine Holidays*

Work in:	Children's summer camps
Dates of jobs:	(6) 15th June – 20th [illegible] June
Staff must be:	(7) [illegible] 19 and 26 years old
Staff must be able to:	(8) fish and cook
Staff will earn:	(9) £ [illegible] 400 per week
Send a letter and:	(10) 187347

Part 1

Questions 1 – 6

For each question, choose the correct answer.

1

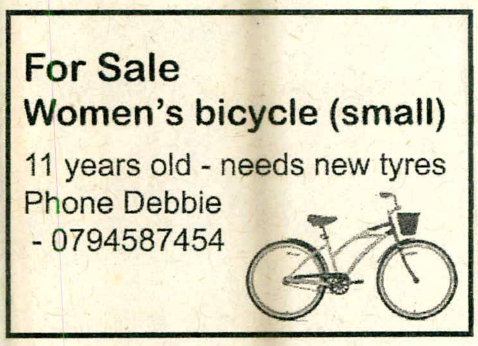

A The bicycle that's for sale was built for a child.

B Some parts of the bicycle must be changed.

C Debbie is selling the bike because she's too big for it now.

2

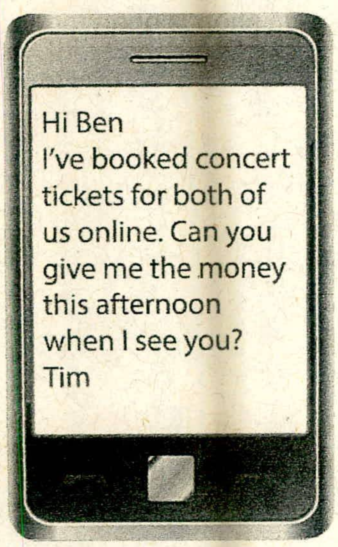

A Tim thinks Ben should look on the concert website.

B Tim hopes that Ben will be able to come with him.

C Tim wants to know if Ben can pay him back today.

3

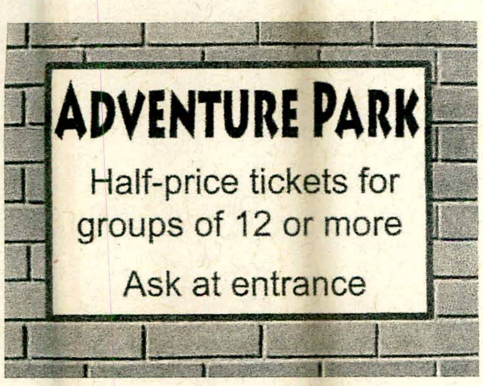

A You get into the park by going this way.

B It is more expensive to go here alone.

C You will have fun if you come with friends.

4

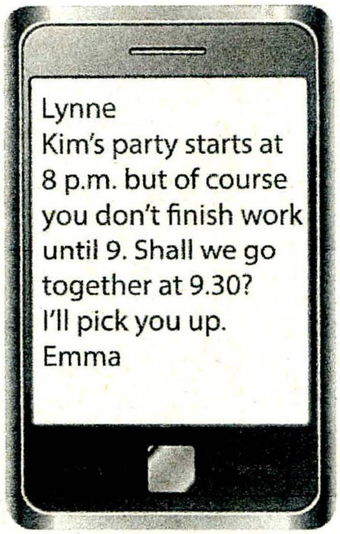

A Emma knows that Lynne can't be at the party when it starts.

B Emma wants to go to the party a bit later than Lynne.

C Emma wants to go out with Lynne but not to the party.

5

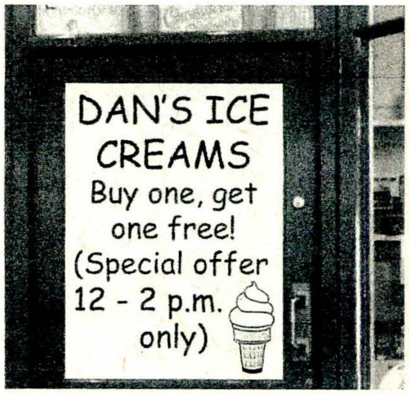

A The ice cream shop is open for only 2 hours.

B Two ice creams will cost the same as one.

C You can get free ice creams all afternoon.

6

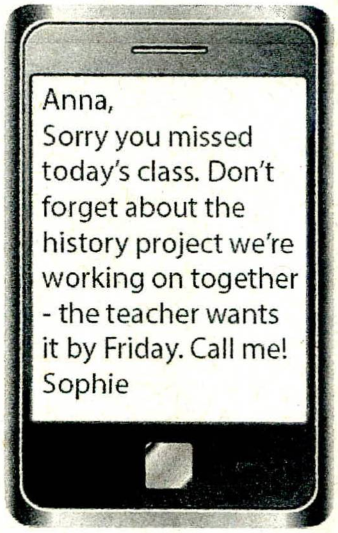

Why did Sophie write this message?

A to check if Anna has completed her homework

B to let Anna know what they did in class today

C to ask Anna to contact her about the homework

Part 3

Questions 11 – 15

For each question, choose the correct answer.

You will hear Robert talking to his friend, Laura, about a trip to Dublin.

11 Who has already decided to go with Robert?

A family members

B colleagues

C tennis partners

12 They'll stay in

A a university.

B a guest house.

C a hotel.

13 Laura must remember to take

A a map.

B a camera.

C a coat.

14 Why does Laura like Dublin?

A The people are friendly.

B The buildings are interesting.

C The shops are beautiful.

15 Robert's excited about the trip to Dublin because

A he can't wait to go to the music festival.

B he loves the food there.

C he wants to go to a new art exhibition.

Turn over ➡

Part 4

Questions 16 – 20

For each question, choose the correct answer.

---

16 You will hear a woman talking to her friend about why she's bought a motorbike.
Why did she buy it?

A It's fast.

B It was cheap.

C It'll be easy to repair.

17 You will hear two friends talking about going to University.
What subject is the man going to study?

A history

B geography

C chemistry

18 You will hear two friends talking about a photograph.
What's the photograph of?

A a sports stadium

B a zoo

C a school playground

19 You will hear a woman talking to a friend on the phone.
Why's she upset?

A Her train was delayed.

B She's lost her wallet.

C She's broken her glasses.

20 You will hear a woman talking to her friend, David, about something she's bought.
What has she bought?

A some clothes

B some food

C some games

Questions 21 – 25

For each question, choose the correct answer.

You will hear Simon talking to Maria about a party.
What will each person bring to the party?

Example

**0**	Maria	B

**People**				**Food**	
**21**	Barbara	☐		**A**	bread
				**B**	cake
**22**	Simon	☐		**C**	cheese
**23**	Anita	☐		**D**	chicken
				**E**	fish
**24**	Peter	☐		**F**	fruit
**25**	Michael	☐		**G**	ice cream
				**H**	salad

You now have 6 minutes to write your answers on the answer sheet.

CAMBRIDGE
English

Key

Reading and Writing

Sample Test

Time 1 hour

INSTRUCTIONS TO CANDIDATES

Do not open this question paper until you are told to do so.

Write your name, centre number and candidate number on your answer sheet if they are not already there.

Read the instructions for each part of the paper carefully.

Answer all the questions.

Read the instructions on the answer sheet.

Write your answers on the answer sheet. Use a pencil.

You **must** complete the answer sheet within the time limit.

At the end of the test, hand in both this question paper and your answer sheet.

Copyright © Cambridge University Press & Assessment 2022

Part 2

Questions 7 – 13

For each question, choose the correct answer.

		Tasha	Danni	Chrissie
7	Who writes both a magazine and a blog?	A	B	C
8	Who says that studying and writing a blog at the same time can be hard?	A	B	C
9	Who answers questions from other people who read her blog?	A	B	C
10	Who plans to stop writing her blog soon?	A	B	C
11	Who didn't have many people reading her blog in the beginning?	A	B	C
12	Who asks a member of her family to help her write her blog?	A	B	C
13	Who says writing a blog is easier than some other types of writing?	A	B	C

Young blog writers

Tasha

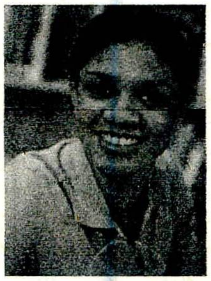

Last year I wrote for my college magazine, which I found really difficult, but I don't think it's hard to write a good blog. Mine is about things from daily life that make me laugh. My older brother also has a blog, but we're writing about different subjects. We don't discuss what we're planning, but we read each other's blogs sometimes. I like giving advice to people who write in asking for it – it's good to know I've helped.

Danni

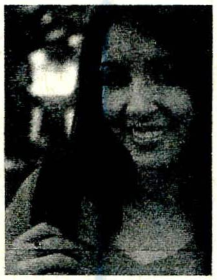

I started writing my popular film blog because I love movies. I like it when readers send me articles by email about a film they've seen, and I put these on my blog for everyone to read. I'm still at college, so I'm careful about spending too long on my blog, which is difficult as writing well takes time. I don't think I'll write it for much longer. I'm busy, and it's time to do something new.

Chrissie

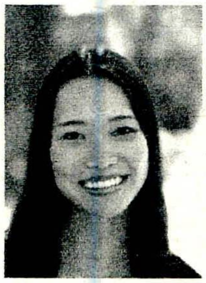

I began writing on a school magazine. I stopped after a few years, but I missed it, so I started my own – I'm still writing it now! The blog's new for me, and I write about daily life. I get ideas from friends or my sister when I can't decide what to write about – we always think of something interesting, sad or serious. At first, almost nobody visited my site, but now more do, I've had some lovely comments.

Question 31

You want to go swimming on Saturday with your English friend, Toni.
Write an email to Toni.

In your email:

- ask Toni to go swimming with you on Saturday
- say where you want to go swimming
- say how you will travel there.

Write **25 words** or more.

Write the email/story on your answer sheet.

hello toni, do you want to come on saterday to swin with me?
we can go swimming in cancun beach
Take a bus to the terminal and I will Pick you up in my car

Question 32

Look at the three pictures.
Write the story shown in the pictures.
Write **35 words** or more.

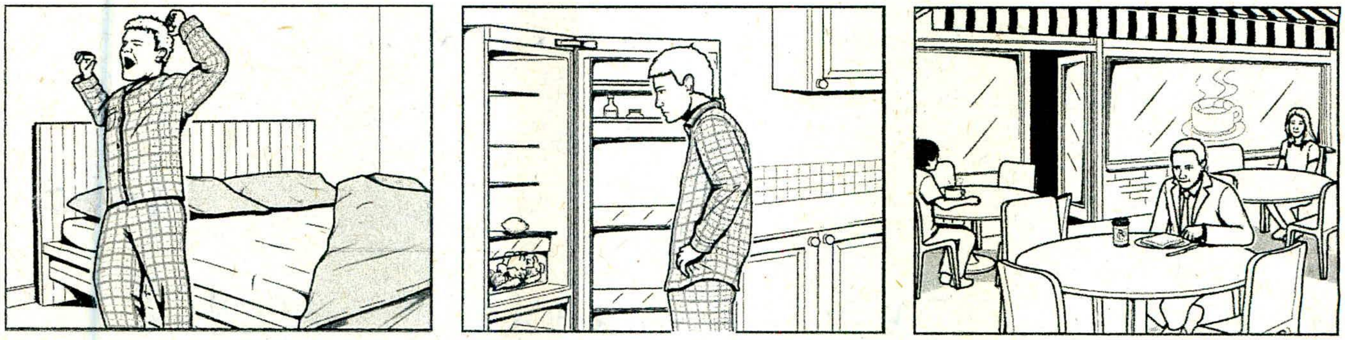

Write the email/story on your answer sheet.

Part 3

Questions 14 – 18

For each question, choose the correct answer.

A family of dancers

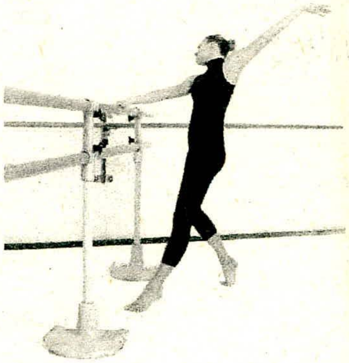

The women in the Watson family are all crazy about ballet. These days, Alice Watson gives ballet lessons, but for many years, she was a dancer with the National Ballet Company. Her mother, Hannah, also had a full-time job there, making costumes for the dancers.

Alice's daughter Demi started learning ballet as soon as she could walk. 'I never taught her,' says Alice, 'because she never let me.' Now aged sixteen, Demi is a member of the ballet company where her mother was the star dancer for many years.

Alice's husband, Jack, is an electrician. They met while he was working at a theatre where she was dancing and got married soon after. 'When Demi started dancing, the house was too small for her and Alice to practise in so I made the garage into a dance studio. Now the living room is nice and quiet when I'm watching television!' he says.

Last month, Demi was invited to dance in the ballet *Swan Lake*. Of course, Alice and Hannah were in the audience and even Jack was there, which made it very special for Demi. Jack says, 'I'm not that interested in ballet myself but it's fantastic seeing Demi taking her first steps with Alice's old company!' Demi was wearing a dress that Hannah made for Alice many years before.

'It was very exciting for all of us,' says Hannah. 'Demi's way of dancing is very like Alice's. I know I'm her grandmother, but I think she has a great future!'

14 What is Alice Watson's job now?

A dancer

B teacher

C dress-maker

15 Demi had her first ballet lessons

A at a very young age.

B at the National Ballet Company.

C from her mother.

16 Jack helped his wife and daughter by

A moving to a larger house.

B letting them use the living room for dancing.

C making a place for them to practise in.

17 What was the best thing about the *Swan Lake* show for Demi?

A It was her first show with the company.

B All her family were there.

C She was wearing a new dress.

18 Hannah says that Demi

A will be a star one day.

B is her favourite granddaughter.

C dances better than Alice did.

Questions 19 – 24

For each question, choose the correct answer.

William Perkin

William Perkin was born in London in 1838. As a child he had many hobbies, including model making and photography. But it was the **(19)**.............. of chemistry that really interested him. At the age of 15, he went to college to study it.

While he was there, he was **(20)** ....to make a medicine from coal. This didn't go well, but when he was working on the problem, he found a cheap **(21)** ....to make the colour purple. At that **(22)**................it was very expensive to make clothes in different colours. William knew he could make a business out of his new colour. Helped by his father and brother, William **(23)** ....his own factory to make the colour. It sold well, and soon purple clothes **(24)** ....very popular in England and the rest of the world.

19	(A)	class	B	subject	C	course
20	A	thinking	(B)	trying	C	deciding
21	(A)	way	B	path	C	plan
22	A	day	(B)	time	C	hour
23	A	brought	B	turned	(C)	opened
24	(A)	began	B	arrived	C	became

Part 9

Questions 25 – 30

For each question, write the correct answer.
Write **one** word for each gap.

Example: | **0** | you |

From:	Maria
To:	John

I hope **(0)**................are well. I'm having a great holiday here in Thailand. Our hotel is very nice and there are a lot of good restaurants near it.

Yesterday morning, we went to **(25)**................ lovely beach. We had to leave before lunch because it was very hot. We went to a party **(26)**................the evening in the centre **(27)**................the town. Everyone had a good time and we got back at midnight. Tomorrow, we want to **(28)** .... on a boat trip or **(29)**................ tennis.

I'll show you my photos **(30)**................ I get back.

See you soon,

Maria
