# Supplementary material for: An activity theory-based exploration of “Eyeland”, a task-based serious game for EFL visually impaired students
Source: PeerJ Comput Sci. 2025 Apr 23;11:e2631. doi: 10.7717/peerj-cs.2631 (PMC12190295; doi:10.7717/peerj-cs.2631)
Supplement: Supplemental Information 7 — Answers shared by students while taking the post-test. [file peerj-cs-11-2631-s007.pdf]

socrative

Name Isabella Ricero
Date 01/06/23

Post test

Score

1. Are you a student?

yes, i'am

2. Where do you live?

i live in Barranquilla

3. Where are you from?

i am from Colombia

4. What is your name?

my name is isabella

5. Do you like rock music?

No, I don't like

6. What time is it?

(A) It is ten after three

(B) It is a quarter past ten

(C) It is ten o' clock

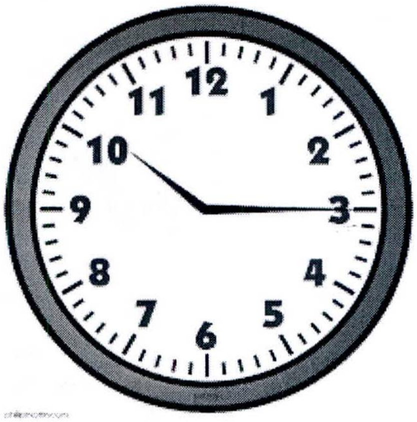

7. When is the party?

(A) It is on July twenty fifth

(B) It is on July twenty five

(C) It is in July twenty fifth

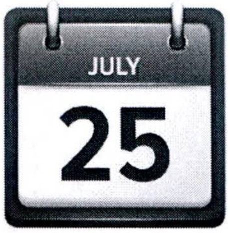

VectorStock®

8. What was the weather like on the picnic?

(A) sunny

(B) windy

(C) rainy

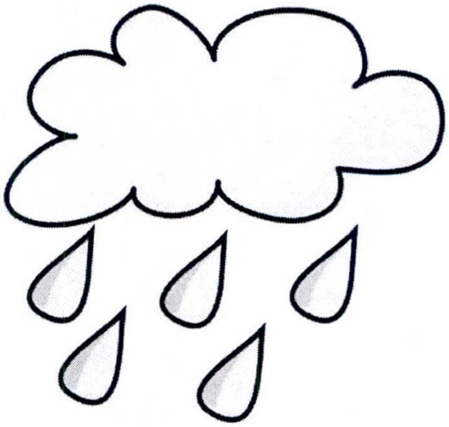

9. How much are the tickets?

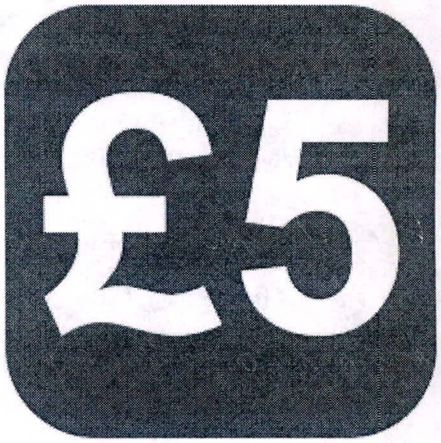

(A) fifteen pound

(B) fifty pounds

(●) five pounds

10. Read the short text and select the best option

-Hi Conor,
I am at the supermarket at the moment. Are there any tomatoes at home? Please let me know.

Kevin.

(A) Kevin wants Conor to buy tomatoes

(B) Kevin thinks that Conor is in the supermarket

(●) Kevin wants to know if there are tomatoes at home

11. Read the short text and select the best option

Adventure Park!
Hal-price tickets for groups of 12 or more. Ask at entrance

(A) You get into the park by going this way

(●) It is more expensive if you go alone

(C) You will have fun if you come with less friends

12. Read the short text and select the best option

Dear Lynn,
The party is in Mary's house at 8 pm. Remember that her house is next to the new bridge. If you come at 7 pm I can pick you up.

Emma.

(●) Emma wants to go to the party with Lynn

(B) Emma lives next to the bridge

(C) Emma wants to go to the party alone

13. You want to go swimming on Saturday with Tony. Write an invitation to Tony.

-Ask Tony to go swimming
-Say where you want to go
-Say how you will travel there

Hello tony, Do you want to go swimming on this saturday? I want to go to the new Pool. Maybe we will go by bus or taxi. i hope your answer, see you later.

14.

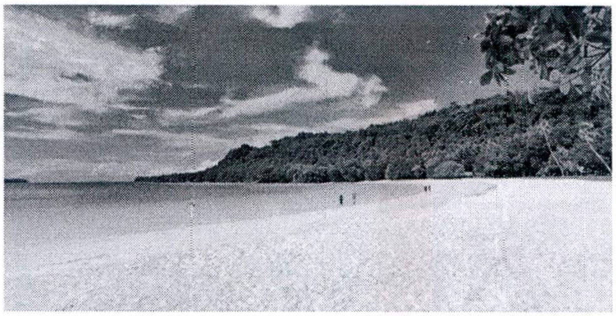

What is the photograph about?

(A) a road
(B) a stadium
(C) a beach

15.

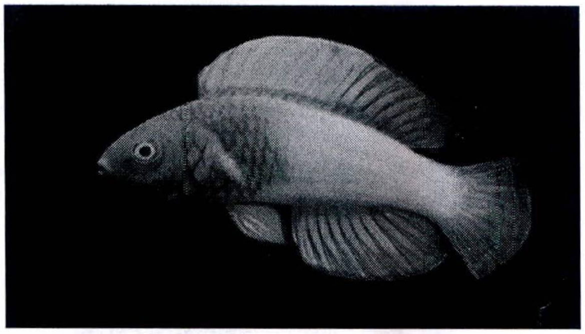

What is the picture about?

(A) fish
(B) dolphin
(C) chicken

socrative

Name tania vizcaino
Date 1/Junio/23

Post test

Score

---

1. Are you a student?

Yes, I am studenT

2. Where do you live?

i live in the santuario

3. Where are you from?

I am from barranquilla

4. What is your name?

my name is tania vizcaino

5. Do you like rock music?

I don't lik rock music

6. What time is it?

(A) It is ten after three

(B) It is a quarter past ten

(C) It is ten o' clock

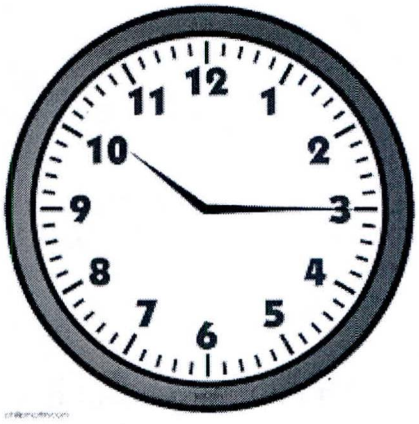

7. When is the party?

(A) It is on July twenty fifth

(B) It is on July twenty five

(C) It is in July twenty fifth

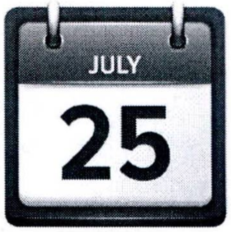

VectorStock®

8. What was the weather like on the picnic?

(A) sunny

(B) windy

(C) rainy

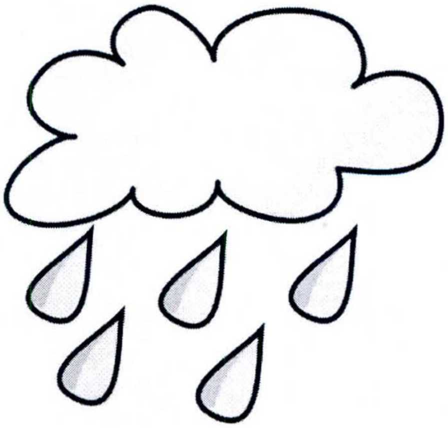

9. How much are the tickets?

(A) fifteen pound

(B) fifty pounds

(C) five pounds

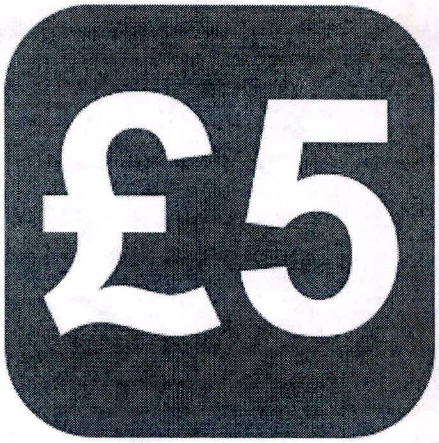

10. Read the short text and select the best option

-Hi Conor,
I am at the supermarket at the moment. Are there any tomatoes at home? Please let me know.

Kevin.

(A) Kevin wants Conor to buy tomatoes

(B) Kevin thinks that Conor is in the supermarket

(C) Kevin wants to know if there are tomatoes at home

11. Read the short text and select the best option

Adventure Park!
Hal-price tickets for groups of 12 or more. Ask at entrance

(A) You get into the park by going this way

(B) It is more expensive if you go alone

(C) You will have fun if you come with less friends

12. Read the short text and select the best option

Dear Lynn,
The party is in Mary's house at 8 pm. Remember that her house is next to the new bridge. If you come at 7 pm I can pick you up.

Emma.

(A) Emma wants to go to the party with Lynn

(B) Emma lives next to the bridge

(C) Emma wants to go to the party alone

13. You want to go swimming on Saturday with Tony. Write an invitation to Tony.

-Ask Tony to go swimming
-Say where you want to go
-Say how you will travel there

14.

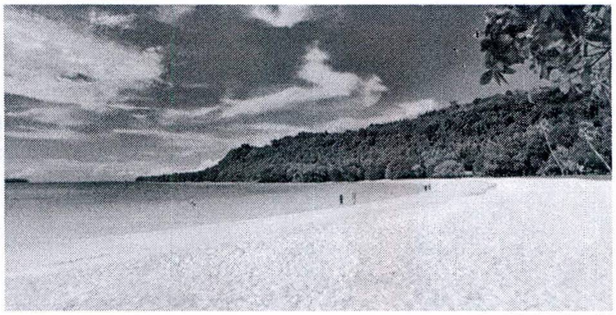

What is the photograph about?

(A) a road
(B) a stadium
(C) a beach

15.

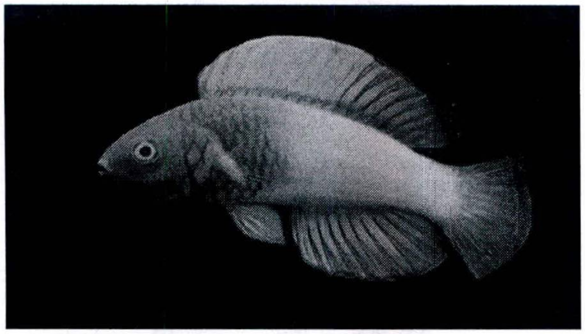

What is the picture about?

(A) fish
(B) dolphin
(C) chicken

socrative

Name Andrea Camacho.
Date Junio 01/23

Post test

Score

(4)

1. Are you a student?

Yes, I am

2. Where do you live?

I live Barranquilla.

3. Where are you from?

I'm from Colombia.

4. What is your name?

Andrea

5. Do you like rock music?

No I don't rock.

Voc

4 buenas

6. What time is it?

(A) It is ten after three

(B) It is a quarter past ten

(C) It is ten o' clock

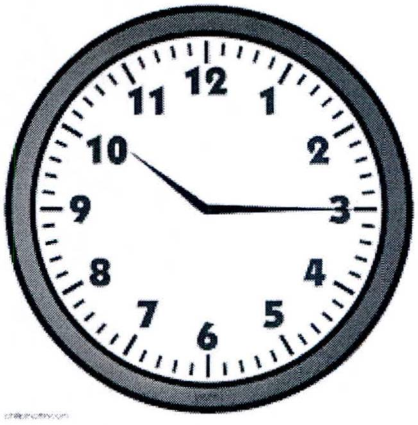

7. When is the party?

(A) It is on July twenty fifth

(B) It is on July twenty five

(C) It is in July twenty fifth

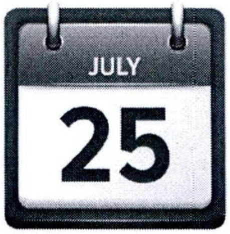

VectorStock®

8. What was the weather like on the picnic?

(A) sunny

(B) windy

(C) rainy

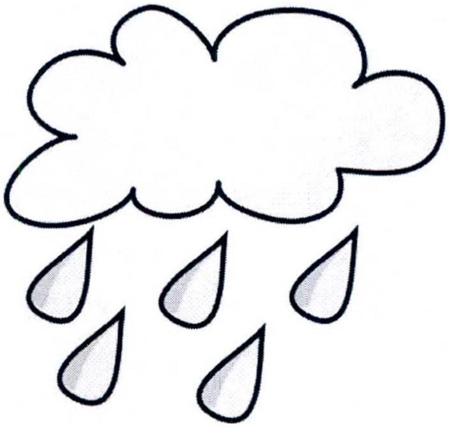

9. How much are the tickets?

(A) fifteen pound

(B) fifty pounds

(C) five pounds

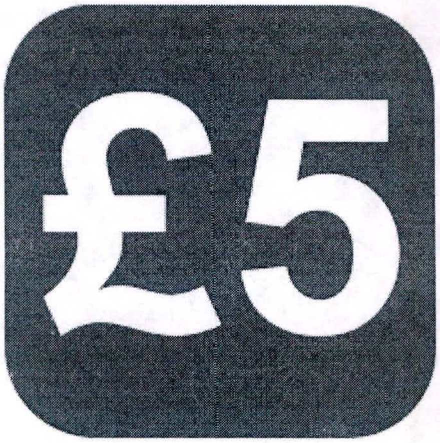

10. Read the short text and select the best option

-Hi Conor,
I am at the supermarket at the moment. Are there any tomatoes at home? Please let me know.

Kevin.

(A) Kevin wants Conor to buy tomatoes

(B) Kevin thinks that Conor is in the supermarket

(C) Kevin wants to know if there are tomatoes at home

11. Read the short text and select the best option

Adventure Park!
Hal-price tickets for groups of 12 or more. Ask at entrance

(A) You get into the park by going this way

(B) It is more expensive if you go alone

(C) You will have fun if you come with less friends

12. Read the short text and select the best option

Dear Lynn,
The party is in Mary's house at 8 pm. Remember that her house is next to the new bridge. If you come at 7 pm I can pick you up.

Emma.

(A) Emma wants to go to the party with Lynn

(B) Emma lives next to the bridge

(C) Emma wants to go to the party alone

13. You want to go swimming on Saturday with Tony. Write an invitation to Tony.

-Ask Tony to go swimming
-Say where you want to go
-Say how you will travel there

Ohh hi Tonny! do you like to swim on is the saturday?
I want to go a new pool
we will go by taxi or walking, See you later.

4 pt

14.

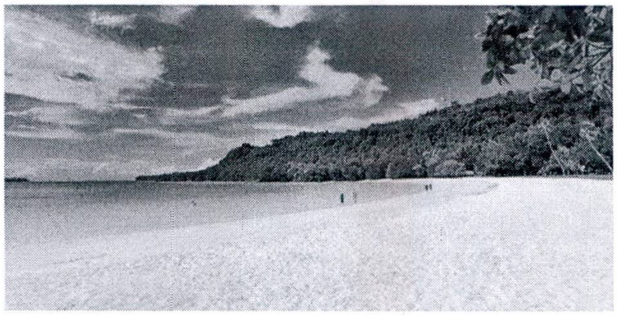

What is the photograph about?

(A) a road
(B) a stadium
(C) a beach

15.

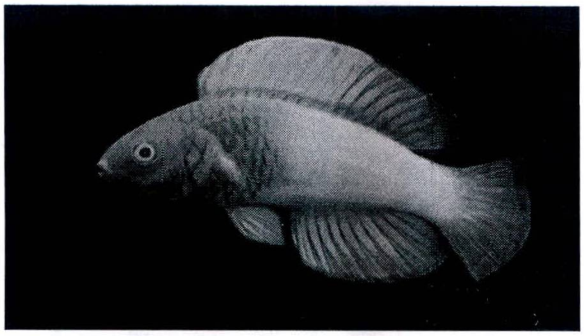

What is the picture about?

(A) fish
(B) dolphin
(C) chicken

socrative

Name Staicey
Date 1/06/23

Post test

Score 10/15

1. Are you a student?

Yes, I'am

2. Where do you live?

I live in barranquilla

3. Where are you from?

I'm from Colombia

4. What is your name?

My name is Staicey Blanco

5. Do you like rock music?

No, I'dont like

Voc

6. What time is it?

(A) It is ten after three

(B) It is a quarter past ten

(C) It is ten o' clock

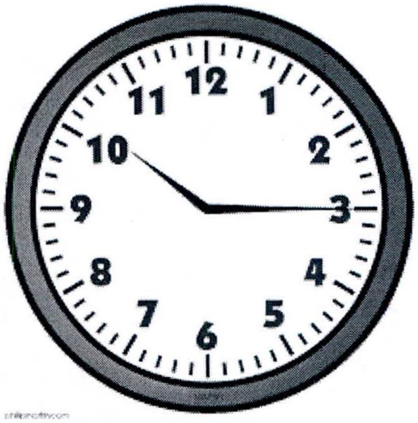

Voc

7. When is the party?

(A) It is on July twenty fifth

(B) It is on July twenty five

(C) It is in July twenty fifth

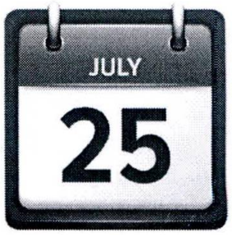

Voc

8. What was the weather like on the picnic?

(A) sunny

(B) windy

(C) rainy

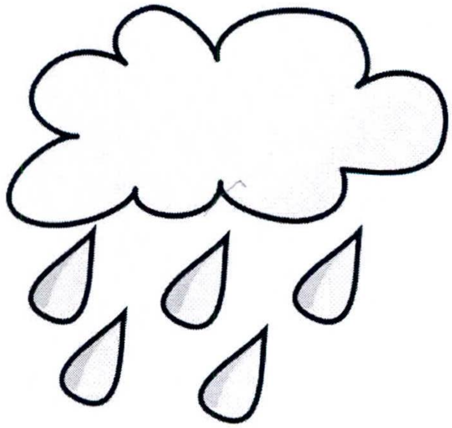

9. How much are the tickets?

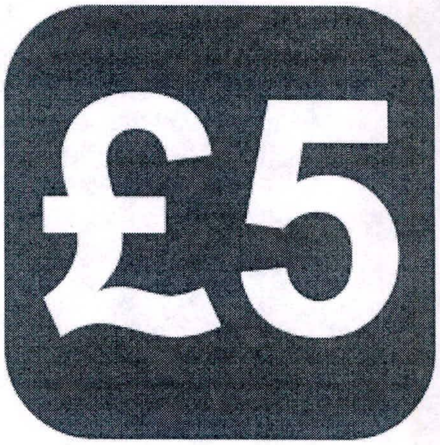

- (A) fifteen pound
- (B) fifty pounds
- (C) five pounds

10. Read the short text and select the best option

-Hi Conor,
I am at the supermarket at the moment. Are there any tomatoes at home? Please let me know.

Kevin.

- (A) Kevin wants Conor to buy tomatoes
- (B) Kevin thinks that Conor is in the supermarket
- (C) Kevin wants to know if there are tomatoes at home

11. Read the short text and select the best option

Adventure Park!
Hal-price tickets for groups of 12 or more. Ask at entrance

- (A) You get into the park by going this way
- (B) It is more expensive if you go alone
- (C) You will have fun if you come with less friends

12. Read the short text and select the best option

Dear Lynn,
The party is in Mary's house at 8 pm. Remember that her house is next to the new bridge. If you come at 7 pm I can pick you up.

Emma.

- (A) Emma wants to go to the party with Lynn
- (B) Emma lives next to the bridge
- (C) Emma wants to go to the party alone

13. You want to go swimming on Saturday with Tony. Write an invitation to Tony.

-Ask Tony to go swimming
-Say where you want to go
-Say how you will travel there

Tony..
on saturday well go to swimming with me.
Where you want to go?

Voc

14.

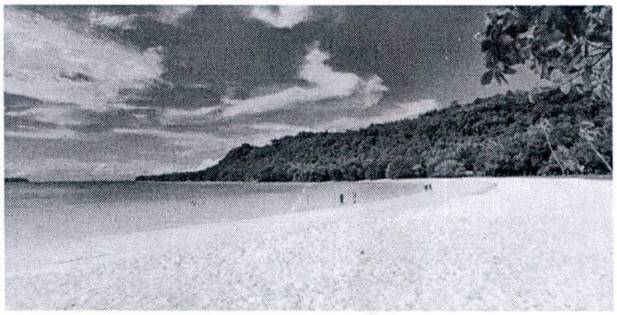

What is the photograph about?

(A) a road
(B) a stadium
(C) a beach

Voc

15.

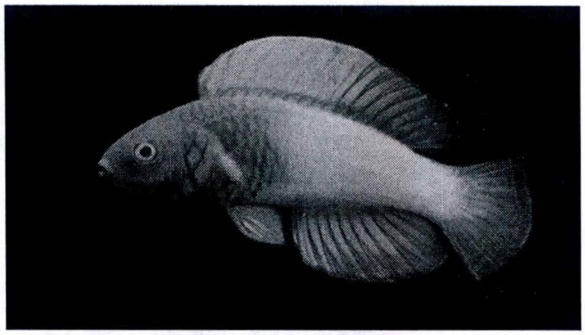

What is the picture about?

(A) fish
(B) dolphin
(C) chicken

socrative

Name Kelvin Enazo
Date 01/06/23

Post test

Score

1. Are you a student?

yes I am student

✓

2. Where do you live?

I live in Barranquilla

✓

3. Where are you from?

I'm From Colombia ✓

4 pts

4. What is your name?

mi name is Kelvin ✓

5. Do you like rock music?

not I'm not like rock

Voc

6. What time is it?

(A) It is ten after three

(B) It is a quarter past ten

(C) It is ten o' clock

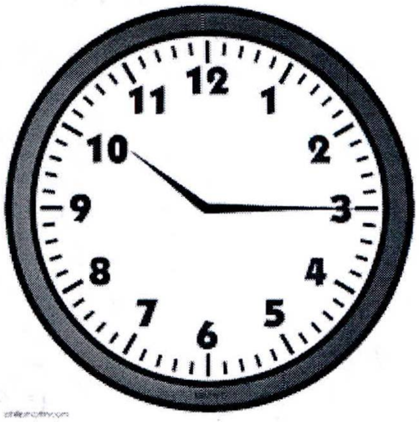

7. When is the party?

(A) It is on July twenty fifth

(B) It is on July twenty five

(C) It is in July twenty fifth

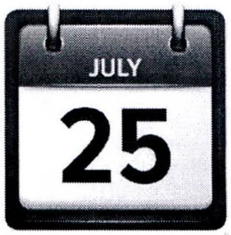

VectorStock

8. What was the weather like on the picnic?

(A) sunny

(B) windy

(C) rainy

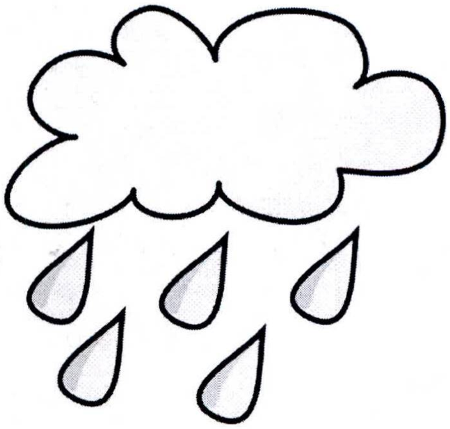

9. How much are the tickets?

(A) fifteen pound

(B) fifty pounds

(C) five pounds

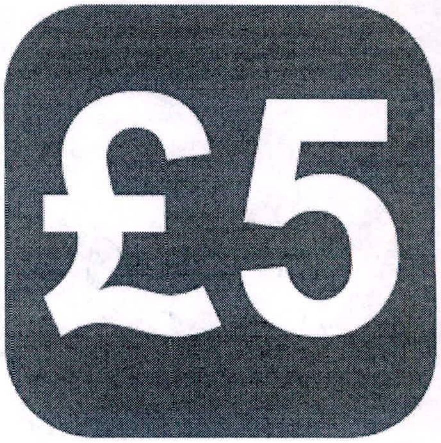

10. Read the short text and select the best option

-Hi Conor,
I am at the supermarket at the moment. Are there any tomatoes at home? Please let me know.

Kevin.

(A) Kevin wants Conor to buy tomatoes

(B) Kevin thinks that Conor is in the supermarket

(C) Kevin wants to know if there are tomatoes at home

11. Read the short text and select the best option

Adventure Park!
Hal-price tickets for groups of 12 or more. Ask at entrance

(A) You get into the park by going this way

(B) It is more expensive if you go alone

(C) You will have fun if you come with less friends

12. Read the short text and select the best option

Dear Lynn,
The party is in Mary's house at 8 pm. Remember that her house is next to the new bridge. If you come at 7 pm I can pick you up.

Emma.

(A) Emma wants to go to the party with Lynn

(B) Emma lives next to the bridge

(C) Emma wants to go to the party alone

13. You want to go swimming on Saturday with Tony. Write an invitation to Tony.

-Ask Tony to go swimming
-Say where you want to go
-Say how you will travel there

Hi tony, can you come swiming on saturday?

14.

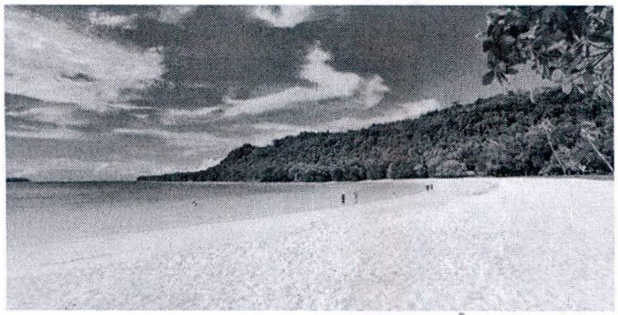

What is the photograph about?

(A) a road
(B) a stadium
(C) a beach

15.

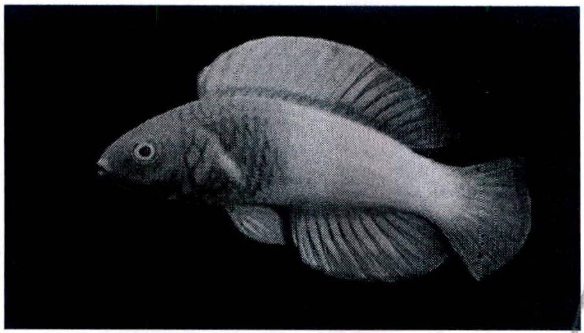

What is the picture about?

(A) fish
(B) dolphin
(C) chicken

socrative

Name

Date

Post test Juan

Score 12/14

---

1. Are you a student?

<yo con el inglés> Yes

2. Where do you live?

in B/quilla

3. Where are you from?

I am from Colombia 4pt

4. What is your name?

Juan

5. Do you like rock music?

No

5pt

Voc

5pts

6. What time is it?

Ⓐ It is ten after three

Ⓑ It is a quarter past ten

Ⓒ It is ten o' clock

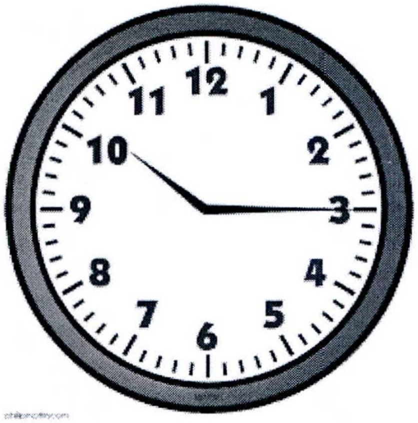

7. When is the party?

Ⓐ It is on July twenty fifth

Ⓑ It is on July twenty five

Ⓒ It is in July twenty fifth

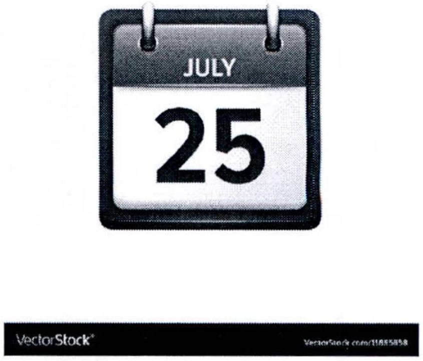

8. What was the weather like on the picnic?

Ⓐ sunny

Ⓑ windy

Ⓒ rainy

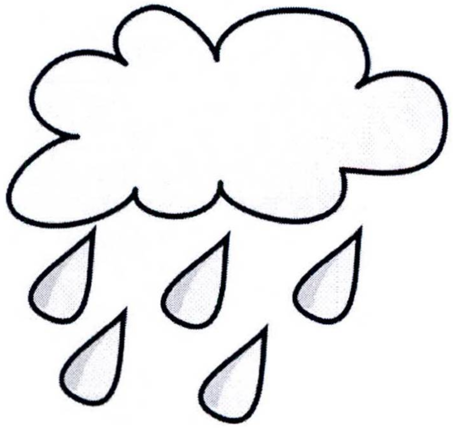

9. How much are the tickets?

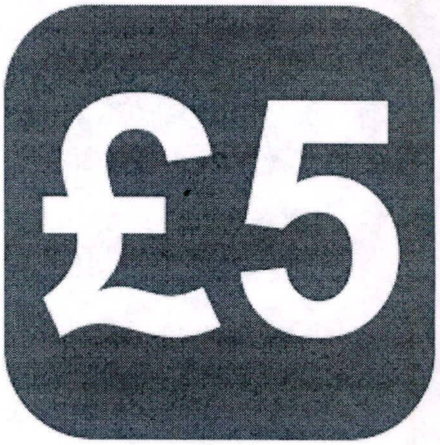

(A) fifteen pound

(B) fifty pounds

(C) five pounds

10. Read the short text and select the best option

-Hi Conor,
I am at the supermarket at the moment. Are there any tomatoes at home? Please let me know.

Kevin.

(A) Kevin wants Conor to buy tomatoes

(B) Kevin thinks that Conor is in the supermarket

(C) Kevin wants to know if there are tomatoes at home

11. Read the short text and select the best option

Adventure Park!
Hal-price tickets for groups of 12 or more. Ask at entrance

(A) You get into the park by going this way

(B) It is more expensive if you go alone

(C) You will have fun if you come with less friends

12. Read the short text and select the best option

Dear Lynn,
The party is in Mary's house at 8 pm. Remember that her house is next to the new bridge. If you come at 7 pm I can pick you up.

Emma.

(A) Emma wants to go to the party with Lynn

(B) Emma lives next to the bridge

(C) Emma wants to go to the party alone

13. You want to go swimming on Saturday with Tony. Write an invitation to Tony.

-Ask Tony to go swimming
-Say where you want to go
-Say how you will travel there

14.

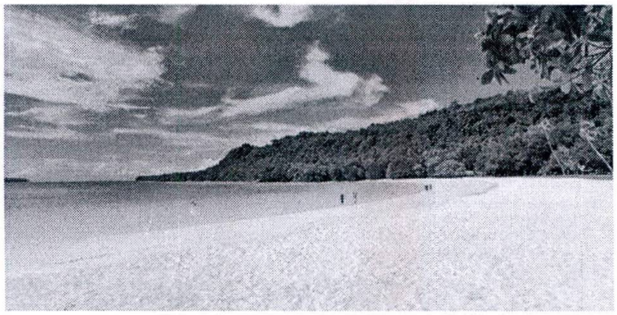

What is the photograph about?

(A) a road

(B) a stadium

(C) a beach

15.

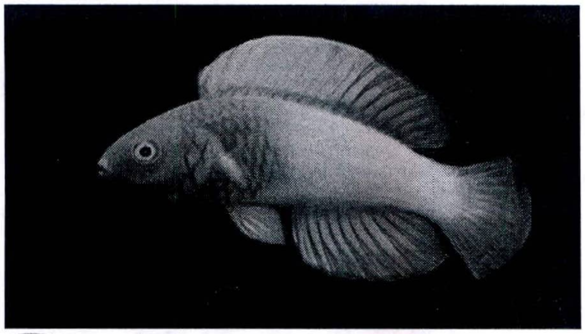

What is the picture about?

(A) fish

(B) dolphin

(C) chicken

socrative

Name Jesus Daniel
Date Lopez Castro
Jonio 1/2023

Post test

Score

4pts

1. Are you a student?

Yes ✓

2. Where do you live?

I live in Barranquilla ✓

3. Where are you from?

I | from Colombia
am

4. What is your name?

My name is Jesus ✓

5. Do you like rock music?

No

Voc

5pts

6. What time is it?

(A) It is ten after three

(B) It is a quarter past ten

(C) It is ten o' clock

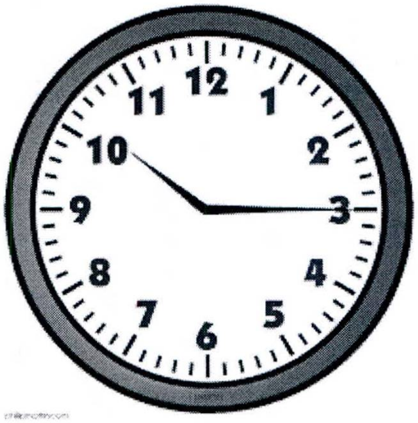

7. When is the party?

(A) It is on July twenty fifth

(B) It is on July twenty five

(C) It is in July twenty fifth

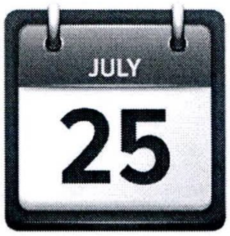

8. What was the weather like on the picnic?

(A) sunny

(B) windy

(C) rainy

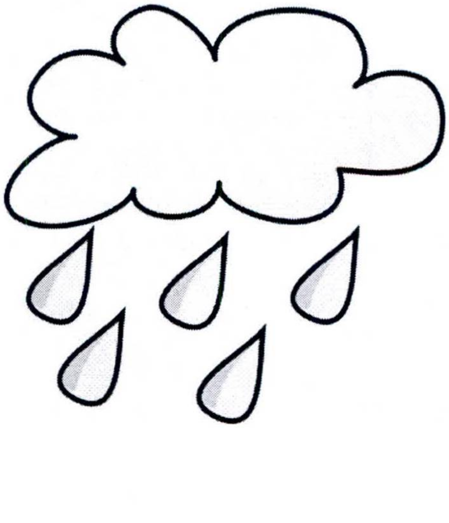

9. How much are the tickets?

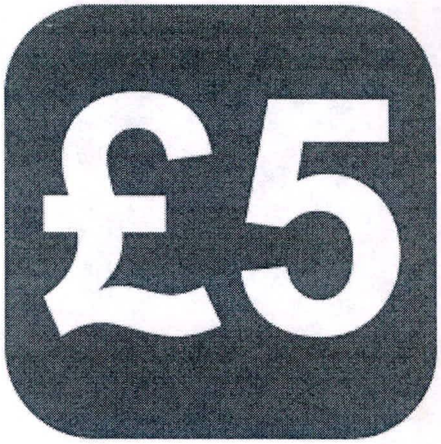

(A) fifteen pound

(B) fifty pounds

(C) five pounds

10. Read the short text and select the best option

-Hi Conor,
I am at the supermarket at the moment. Are there any tomatoes at home? Please let me know.

Kevin.

(A) Kevin wants Conor to buy tomatoes

(B) Kevin thinks that Conor is in the supermarket

(C) Kevin wants to know if there are tomatoes at home

11. Read the short text and select the best option

Adventure Park!
Hal-price tickets for groups of 12 or more. Ask at entrance

(A) You get into the park by going this way

(B) It is more expensive if you go alone

(C) You will have fun if you come with less friends

12. Read the short text and select the best option

Dear Lynn,
The party is in Mary's house at 8 pm. Remember that her house is next to the new bridge. If you come at 7 pm I can pick you up.

Emma.

(A) Emma wants to go to the party with Lynn

(B) Emma lives next to the bridge

(C) Emma wants to go to the party alone

13. You want to go swimming on Saturday with Tony. Write an invitation to Tony.

-Ask Tony to go swimming
-Say where you want to go
-Say how you will travel there

14.

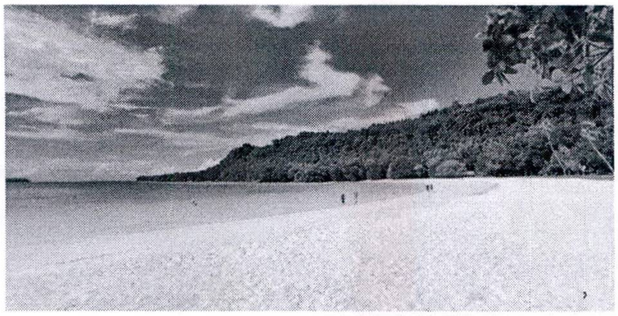

What is the photograph about?

(A) a road

(B) a stadium

(C) a beach

15.

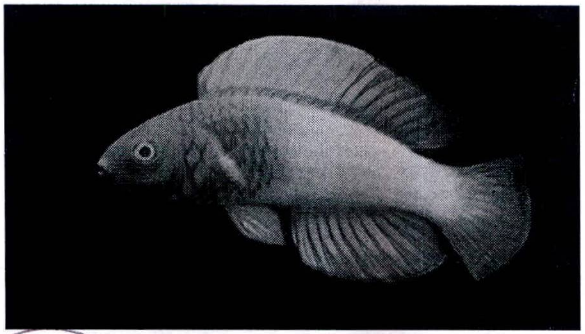

What is the picture about?

(A) fish

(B) dolphin

(C) chicken

socrative

Post test

Name Andres Noguera
Date June 01

Score 10/15

4pts

1. Are you a student?

yes, (i) am student.

2. Where do you live?

I live in Barranquilla.

3. Where are you from?

I am from Colombia

4. What is your name?

My name is Andres

5. Do you like rock music?

I am not.
X

You 1/4 pts

6. What time is it?

(A) It is ten after three
(B) It is a quarter past ten
(C) It is ten o' clock X

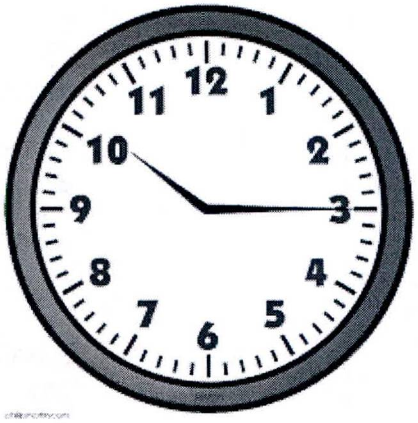

7. When is the party?

(A) It is on July twenty fifth
(B) It is on July twenty five
(C) It is in July twenty fifth X

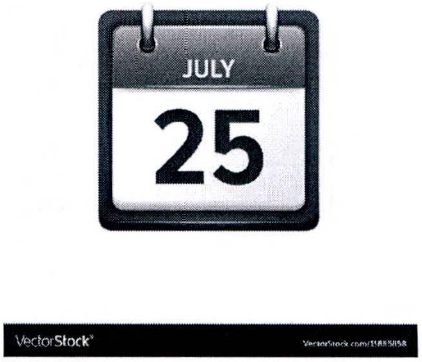

8. What was the weather like on the picnic?

(A) sunny
(B) windy
(C) rainy ✓

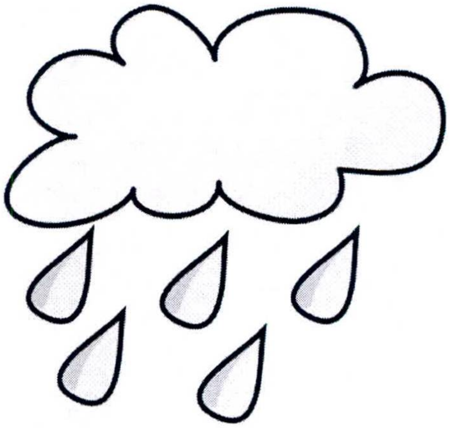

9. How much are the tickets?

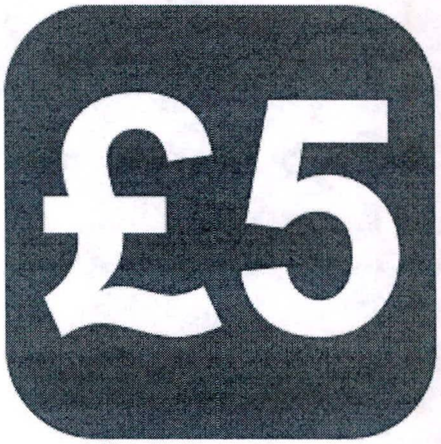

(A) fifteen pound

(B) fifty pounds

(C) five pounds

10. Read the short text and select the best option

-Hi Conor,
I am at the supermarket at the moment. Are there any tomatoes at home? Please let me know.

Kevin.

(A) Kevin wants Conor to buy tomatoes

(B) Kevin thinks that Conor is in the supermarket

(C) Kevin wants to know if there are tomatoes at home

11. Read the short text and select the best option

Adventure Park!
Hal-price tickets for groups of 12 or more. Ask at entrance

(A) You get into the park by going this way

(B) It is more expensive if you go alone

(C) You will have fun if you come with less friends

12. Read the short text and select the best option

Dear Lynn,
The party is in Mary's house at 8 pm. Remember that her house is next to the new bridge. If you come at 7 pm I can pick you up.

Emma.

(A) Emma wants to go to the party with Lynn

(B) Emma lives next to the bridge

(C) Emma wants to go to the party alone

13. You want to go swimming on Saturday with Tony. Write an invitation to Tony.

-Ask Tony to go swimming
-Say where you want to go
-Say how you will travel there

14.

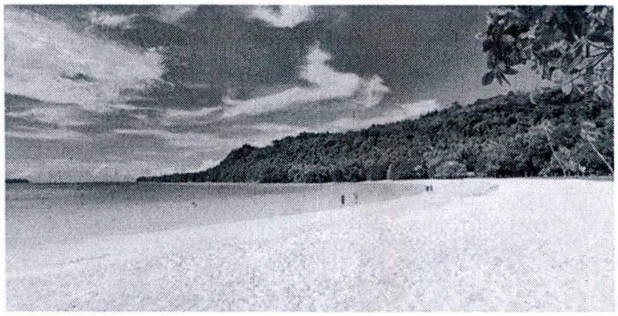

What is the photograph about?

(A) a road

(B) a stadium

(C) a beach

15.

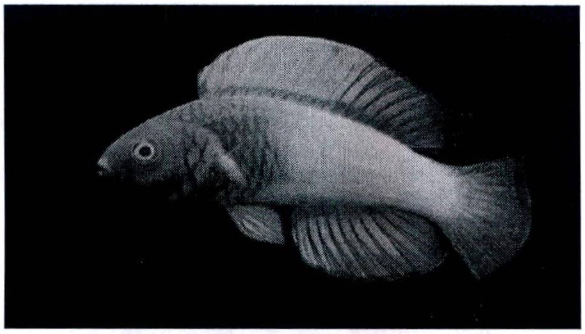

What is the picture about?

(A) fish

(B) dolphin

(C) chicken

socrative

Name Yancelis Gonzalez
Date 01/06/23

Post test

Score 13/15

1. Are you a student?

Yes, I am

4pts

2. Where do you live?

I live in Barranquilla

3. Where are you from?

I am From colombia

4. What is your name?

My name is Yancelis

5. Do you like rock music?

NO, I don'T ~~like~~ rock

6. What time is it?

(A) It is ten after three

(B) It is a quarter past ten ●

(C) It is ten o' clock

✓

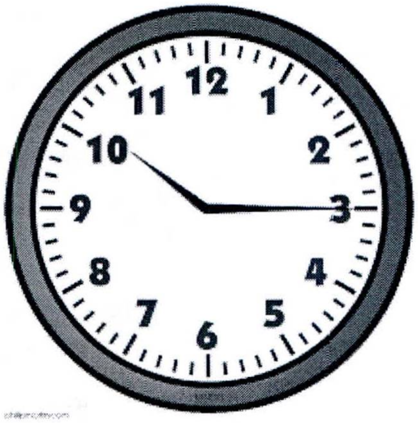

7. When is the party?

(A) It is on July twenty fifth

(B) It is on July twenty five ●

(C) It is in July twenty fifth

✗

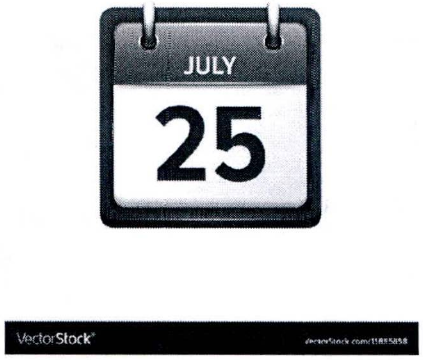

8. What was the weather like on the picnic?

(A) sunny

(B) windy

(C) rainy ●

✓

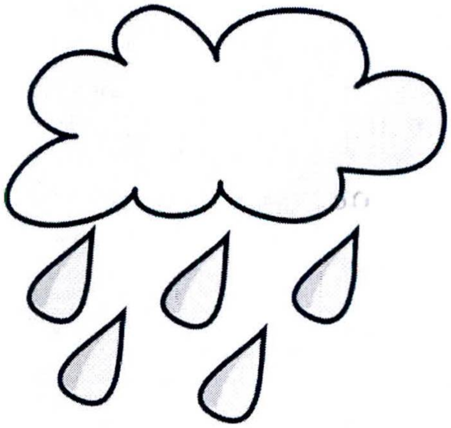

9. How much are the tickets?

Ⓐ fifteen pound

Ⓑ fifty pounds

● five pounds

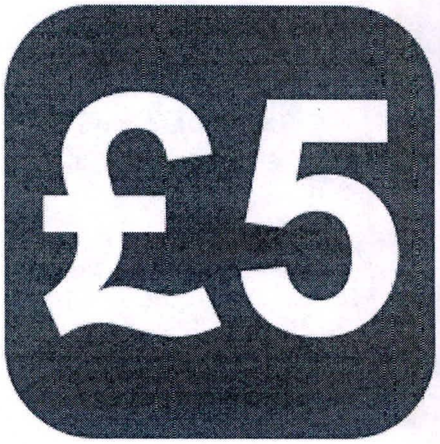

10. Read the short text and select the best option

10pt

-Hi Conor,
I am at the supermarket at the moment. Are there any tomatoes at home? Please let me know.

Kevin.

Ⓐ Kevin wants Conor to buy tomatoes

Ⓑ Kevin thinks that Conor is in the supermarket

● Kevin wants to know if there are tomatoes at home

11. Read the short text and select the best option

Adventure Park!
Hal-price tickets for groups of 12 or more. Ask at entrance

Ⓐ You get into the park by going this way

● It is more expensive if you go alone

Ⓒ You will have fun if you come with less friends

12. Read the short text and select the best option

Dear Lynn,
The party is in Mary's house at 8 pm. Remember that her house is next to the new bridge. If you come at 7 pm I can pick you up.

Emma.

● Emma wants to go to the party with Lynn

Ⓑ Emma lives next to the bridge

Ⓒ Emma wants to go to the party alone

13. You want to go swimming on Saturday with Tony. Write an invitation to Tony.

-Ask Tony to go swimming
-Say where you want to go
-Say how you will travel there

Hello, Tony DO YOU swimming To do on This saTurday? I want TO go To THe Pool

4pts

14.

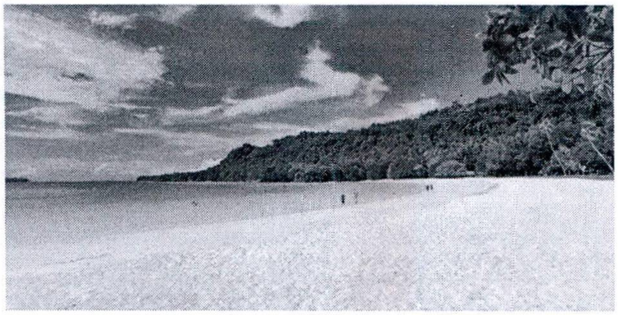

What is the photograph about?

(A) a road
(B) a stadium
(C) a beach

15.

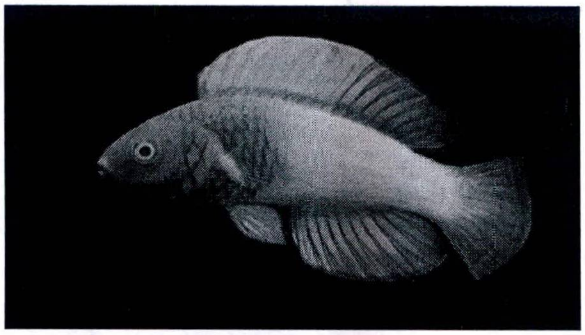

What is the picture about?

(A) fish
(B) dolphin
(C) chicken

socrative

Name Fabian
Date 1st June /2023

Post test

Score 11/15

1. Are you a student?

Yes, iam

2. Where do you live?

I live in Barranquilla

3. Where are you from?

I'm From Colombia

4. What is your name?

My name is Fabian Pacheco N.

5pts

5. Do you like rock music?

No, I don't ✓

Voc

6. What time is it?

● It is ten after three

(B) It is a quarter past ten

(C) It is ten o' clock

✗

4pts

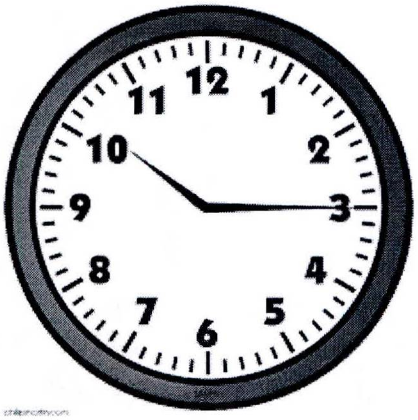

7. When is the party?

(A) It is on July twenty fifth

● It is on July twenty five

(C) It is in July twenty fifth

✗

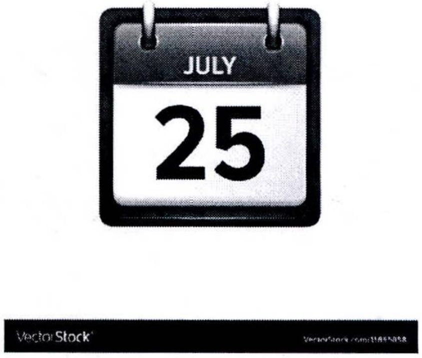

8. What was the weather like on the picnic?

(A) sunny

(B) windy

● rainy

✓

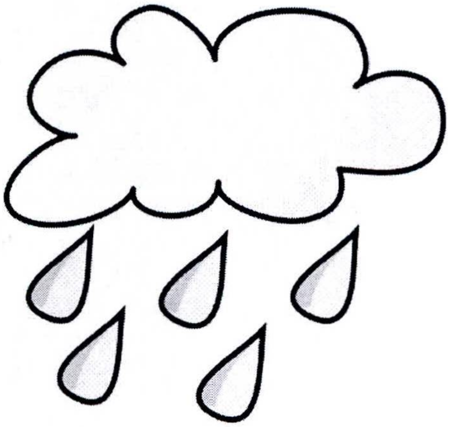

9. How much are the tickets?

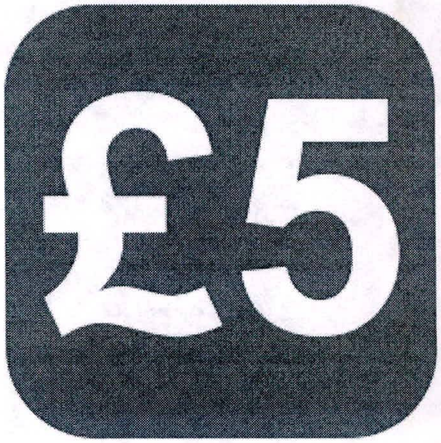

(A) fifteen pound

(B) fifty pounds

● five pounds

10. *Read the* short text and select the best option

-Hi Conor,
I am at the supermarket at the moment. Are there any tomatoes at home? Please let me know.

Kevin.

● Kevin wants Conor to buy tomatoes

(B) Kevin thinks that Conor is in the supermarket

(C) Kevin wants to know if there are tomatoes at home

11. Read the short text and select the best option

Adventure Park!
Hal-price tickets for groups of 12 or more. Ask at entrance

(A) You get into the park by going this way

● It is more expensive if you go alone

(C) You will have fun if you come with less friends

12. Read the short text and select the best option

Dear Lynn,
The party is in Mary's house at 8 pm. Remember that her house is next to the new bridge. If you come at 7 pm I can pick you up.

Emma.

● Emma wants to go to the party with Lynn

(B) Emma lives next to the bridge

(C) Emma wants to go to the party alone

13. You want to go swimming on Saturday with Tony. Write an invitation to Tony.

-Ask Tony to go swimming
-Say where you want to go
-Say how you will travel there

8

Dear Tony
On Saturday we'll go ~~to~~ swiming so let me know if you want ~~go~~ come too with ~~us~~ me I'm waiting your answer

14.

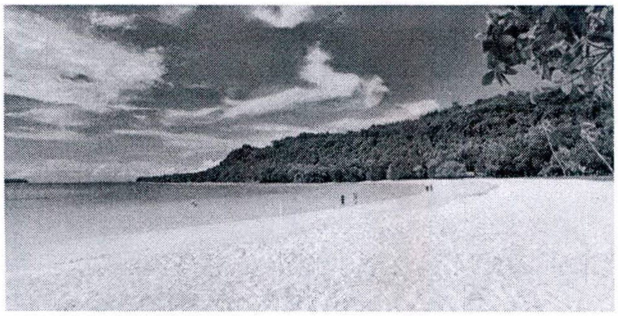

What is the photograph about?

(A) a road
(B) a stadium
(C) a beach

15.

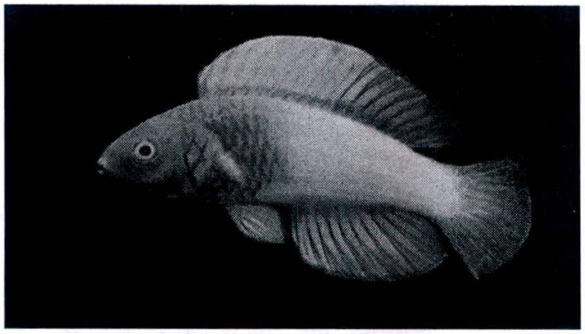

What is the picture about?

(A) fish
(B) dolphin
(C) chicken

socrative

Name Maria Agucoro
Date 1/06/23

Post test

Score

1. Are you a student?

Yes I do

2. Where do you live?

I live in b/quilla Colombia

3. Where are you from?

I'm from Colombia

4. What is your name?

My name is Maria Jose

5. Do you like rock music?

NO

6. What time is it?

(A) It is ten after three

(B) It is a quarter past ten

(C) It is ten o' clock

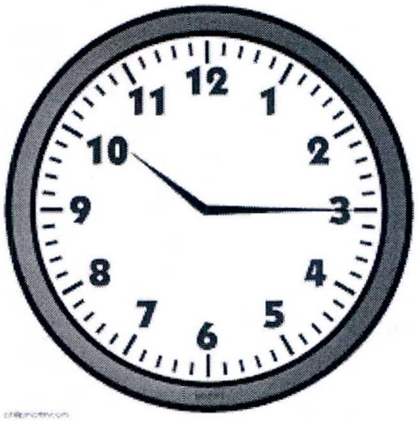

7. When is the party?

(A) It is on July twenty fifth

(B) It is on July twenty five

(C) It is in July twenty fifth

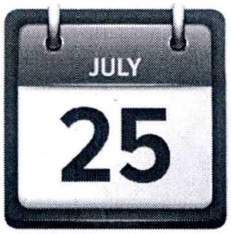

VectorStock®

8. What was the weather like on the picnic?

(A) sunny

(B) windy

(C) rainy

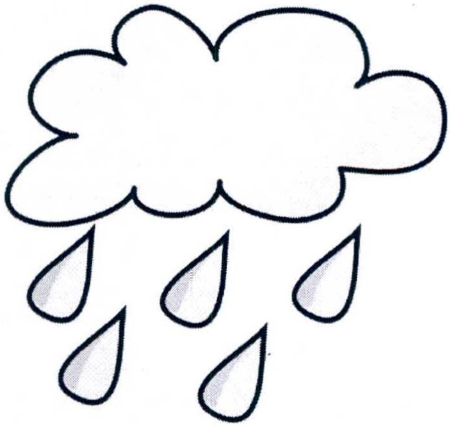

9. How much are the tickets?

(A) fifteen pound

(B) fifty pounds

(C) five pounds

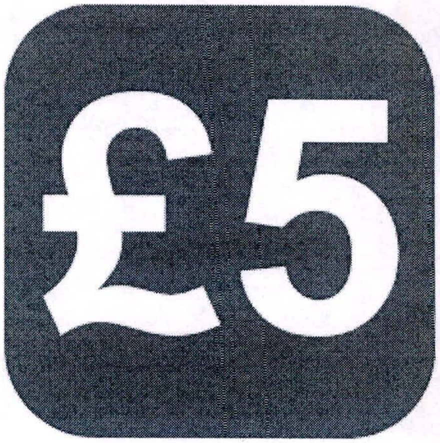

10. Read the short text and select the best option

-Hi Conor,
I am at the supermarket at the moment. Are there any tomatoes at home? Please let me know.

Kevin.

(A) Kevin wants Conor to buy tomatoes

(B) Kevin thinks that Conor is in the supermarket

(C) Kevin wants to know if there are tomatoes at home

11. Read the short text and select the best option

Adventure Park!
Hal-price tickets for groups of 12 or more. Ask at entrance

(A) You get into the park by going this way

(B) It is more expensive if you go alone

(C) You will have fun if you come with less friends

12. Read the short text and select the best option

Dear Lynn,
The party is in Mary's house at 8 pm. Remember that her house is next to the new bridge. If you come at 7 pm I can pick you up.

Emma.

(A) Emma wants to go to the party with Lynn

(B) Emma lives next to the bridge

(C) Emma wants to go to the party alone

13. You want to go swimming on Saturday with Tony. Write an invitation to Tony.

-Ask Tony to go swimming
-Say where you want to go
-Say how you will travel there

14.

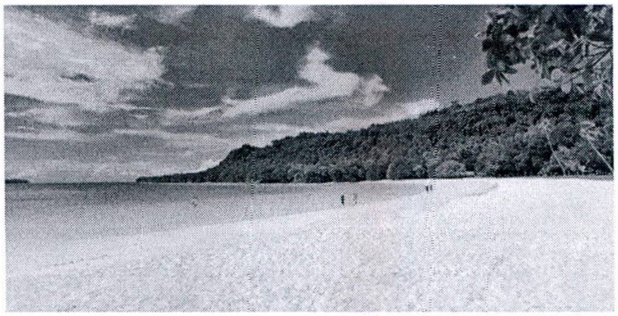

What is the photograph about?

(A) a road

(B) a stadium

(C) a beach

15.

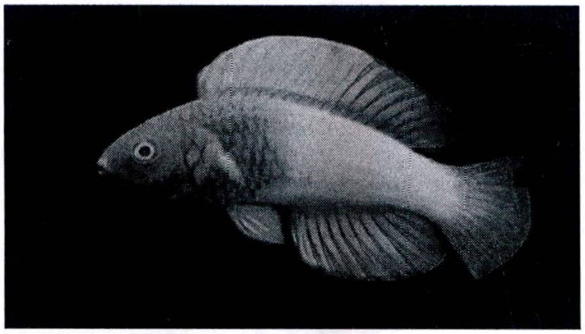

What is the picture about?

(A) fish

(B) dolphin

(C) chicken
