# Supplementary figures and images for: An activity theory-based exploration of “Eyeland”, a task-based serious game for EFL visually impaired students
Source: PeerJ Comput Sci. 2025 Apr 23;11:e2631. doi: 10.7717/peerj-cs.2631 (PMC12190295; doi:10.7717/peerj-cs.2631)

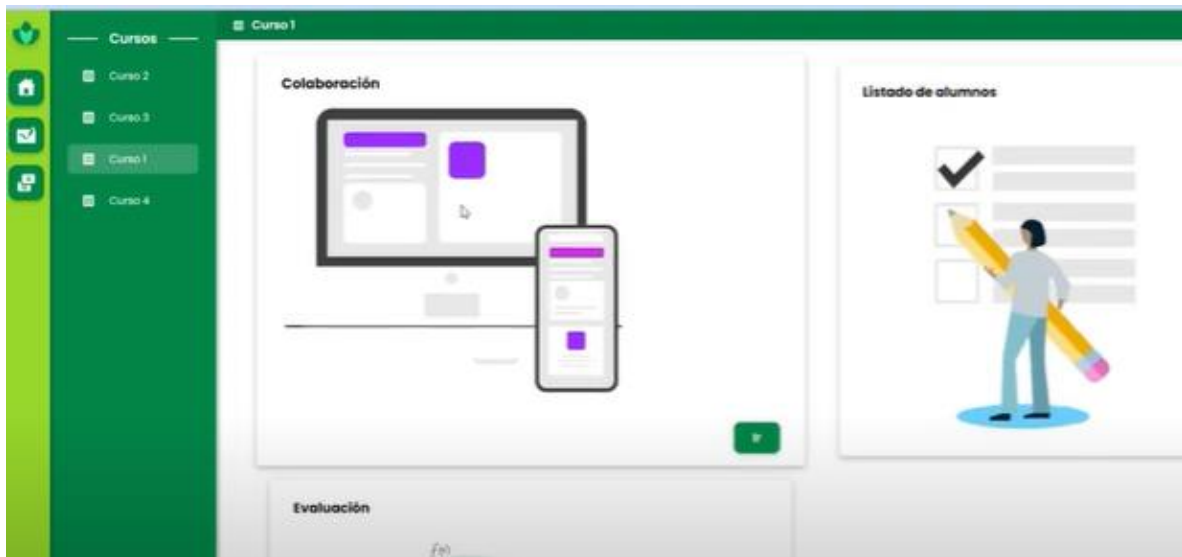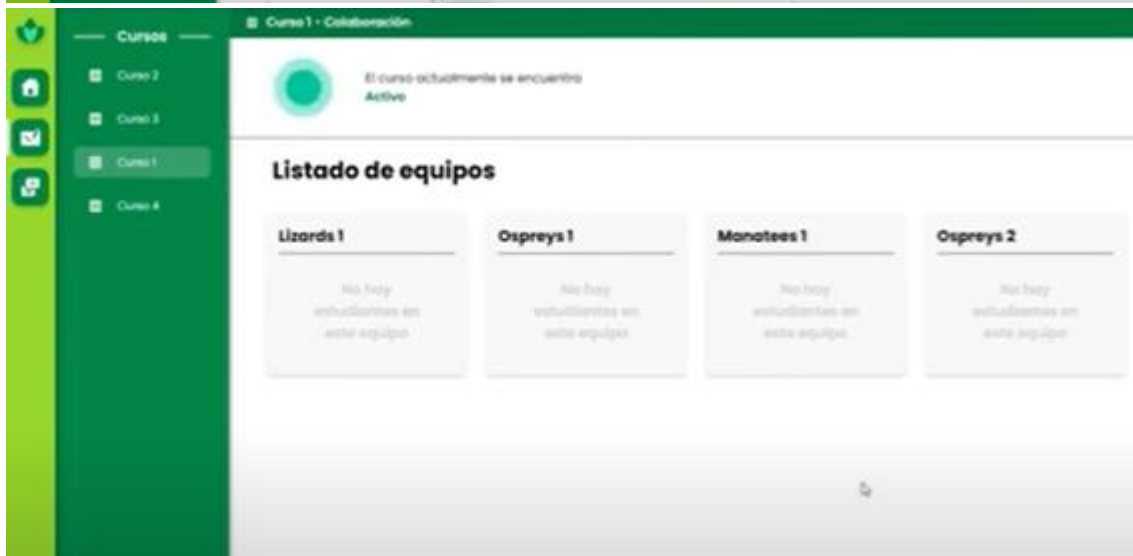

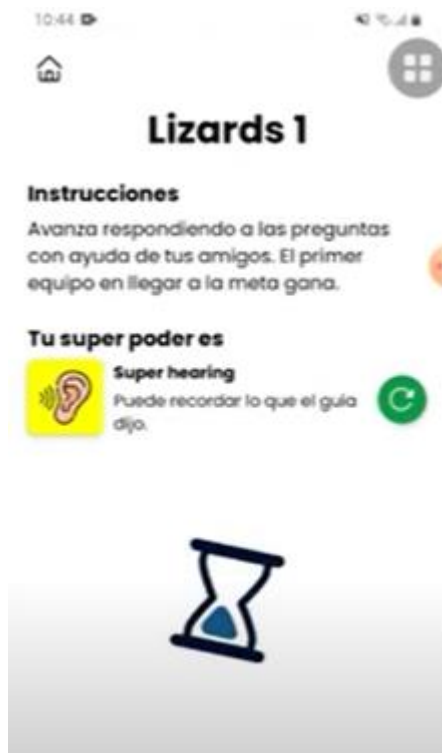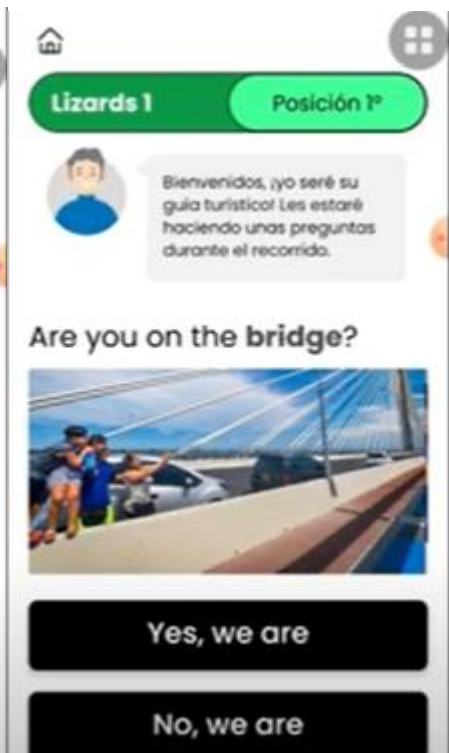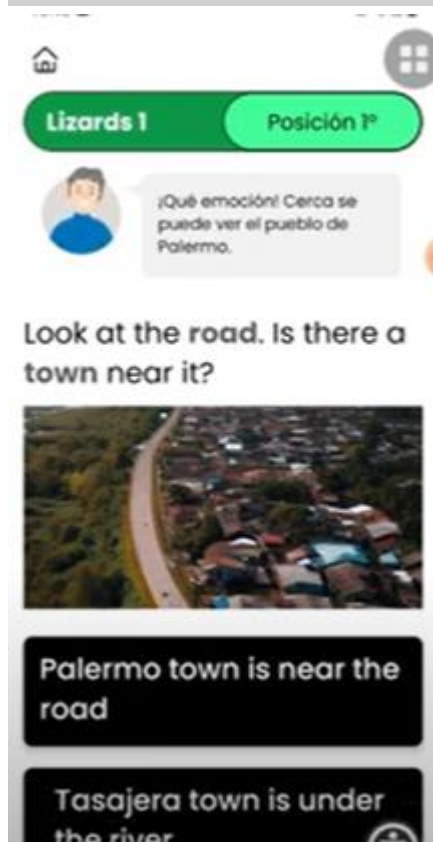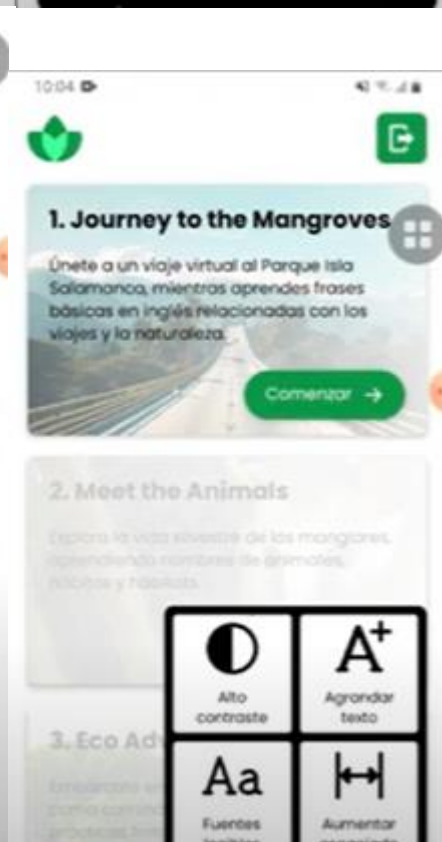

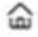

Selecciona la opción correcta

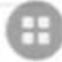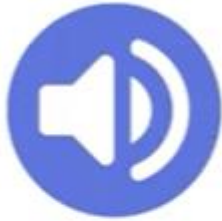

Bridge

Toll

Farm

Supplement: Supplemental Information 8 — Different stages of the app interaction. [file peerj-cs-11-2631-s008.pdf]
